# Comprehensive Characterization of CK1δ-Mediated Tau Phosphorylation in Alzheimer’s Disease

**DOI:** 10.3389/fmolb.2022.872171

**Published:** 2022-06-27

**Authors:** Aileen Roth, Annabelle Sander, Marleen Silke Oswald, Fabian Gärtner, Uwe Knippschild, Joachim Bischof

**Affiliations:** Department of General and Visceral Surgery, University Medical Center Ulm, University of Ulm, Ulm, Germany

**Keywords:** Alzheimer’s disease, AD, casein kinase 1δ, CK1δ, tau phosphorylation, tau aggregation

## Abstract

A main pathological event in Alzheimer’s disease is the generation of neurofibrillary tangles originating from hyperphosphorylated and subsequently aggregated tau proteins. Previous reports demonstrated the critical involvement of members of the protein kinase family CK1 in the pathogenesis of Alzheimer’s disease by hyperphosphorylation of tau. However, precise mechanisms and effects of CK1-mediated tau phosphorylation are still not fully understood. In this study, we analyzed recombinant tau441 phosphorylated by CK1δ *in vitro* via mass spectrometry and identified ten potential phosphorylation sites, five of them are associated to Alzheimer’s disease. To confirm these results, *in vitro* kinase assays and two-dimensional phosphopeptide analyses were performed with tau441 phosphomutants confirming Alzheimer’s disease-associated residues Ser68/Thr71 and Ser289 as CK1δ-specific phosphorylation sites. Treatment of differentiated human neural progenitor cells with PF-670462 and Western blot analysis identified Ser214 as CK1δ-targeted phosphorylation site. The use of an *in vitro* tau aggregation assay demonstrated a possible role of CK1δ in tau aggregation. Results obtained in this study highlight the potential of CK1δ to be a promising target in the treatment of Alzheimer’s disease.

## Introduction

Alzheimer’s disease (AD) is a progressive neurodegenerative disorder characterized by an irreversible process of changes involving specific neurons of the neocortex, hippocampus, and other regions of the brain, leading to a cognitive impairment followed by a mental and functional decline. Generally, AD is responsible for more than 80% of dementia cases in elderly people worldwide (Anand et al., 2014). One of the main neuropathological characteristics is the presence of intraneuronal aggregated neurofibrillary tangles (NFTs) assembled from paired helical filaments (PHF) composed of highly phosphorylated tau proteins (Buée et al., 2000; Kumar and Dogra, 2008).

Tau proteins belong to the family of microtubule-associated proteins (MAPs) (Weingarten et al., 1975) and occur mainly in the axonal compartment of neurons (Binder et al., 1985). In the central nervous system, six alternatively spliced tau isoforms in a range from 352 to 441 amino acids were identified (Goedert et al., 1989), that regulate microtubule assembly by modulating the functional organization of neurons, particularly in growth, polarity and axonal morphology (Buée et al., 2000). Extensive tau phosphorylation at various amino acid residues converts soluble tau proteins into PHF leading to the development of NFTs, which cause tau pathologies in AD and other tauopathies (Grundke-Iqbal et al., 1986; Kosik et al., 1986; Brion et al., 1991). A wide range of proline-directed kinases (e.g., glycogen synthase kinase 3 β (GSK3β) (Llorens-Martín et al., 2014), cyclin-dependent kinase 5 (CDK5) (Kimura et al., 2014)), nonproline-directed kinases (e.g., tau-tubulin kinases (TTBK) (Tomizawa et al., 2001)), microtubule affinity regulated kinases (e.g., MARK) (Matenia and Mandelkow, 2009) and tyrosine kinases (e.g., Fyn and Abl (Lee et al., 2004; Derkinderen et al., 2005)) have been found to phosphorylate tau and contribute to the pathophysiological hallmark of AD.

Potential kinases catalyzing the hyperphosphorylation of tau in AD also include members of the CK1 (formerly named casein kinase 1) family. Members of the CK1 family are highly conserved serine/threonine-specific, ubiquitously expressed protein kinases. So far, seven different CK1 isoforms (α, β, γ1-3, δ and ε) and their splice variants were identified in mammals. CK1 is able to recognize canonical as well as noncanonical consensus sequences within a substrate resulting in over 150 different *in vitro* and *in vivo* substrates (reviewed in Knippschild et al., 2014). The role of CK1 as a potential kinase phosphorylating tau has become of particular interest, because it has been reported that levels of CK1δ were elevated by a factor of 30 in the hippocampus in the brain of AD patients compared with equivalent controls (Ghoshal et al., 1999). Additionally, CK1α and CK1δ have been shown to be tightly associated with neurofibrillary lesions of AD, further implicating CK1 in PHF formation (Kuret et al., 1997; Schwab et al., 2000). CK1δ site-specific tau phosphorylation was detected at Ser202/Thr205 and Ser396/Ser404 in non-neuronal human embryonic kidney 293 (HEK293) cells using immunodetection (Li et al., 2004). In a more comprehensive study, various CK1δ-specific phosphorylation sites were detected analyzing recombinant tau, which was phosphorylated by CK1δ *in vitro* by using mass spectrometry (MS). MS analysis revealed 33 CK1δ-specific tau phosphorylation sites, while previously detected phosphorylation of Ser202/Thr205 and Ser396/Ser404 could not be confirmed (Hanger et al., 2007). In both studies, CK1δ-specific phosphorylation of tau was verified by treating HEK293 or rat cortical neurons with IC261, which was later observed to induce CK1δ- and ε-independent cytotoxic effects by its binding to tubulin leading to microtubule polymerization (Cheong and Virshup, 2011; Stöter et al., 2014; Xian et al., 2021). Because of qualitative methods used in these studies, connection between CK1-mediated tau phosphorylation identified by MS analysis or immunological methods and the role of CK1 in AD by determining the effect of CK1-mediated phosphorylation on tau aggregation has remained elusive.

Tau hyperphosphorylation and tau-phosphorylating kinases have become attractive targets in the treatment of AD. Therefore, the characterization of tau hyperphosphorylation by tau-targeting kinases, such as CK1, is necessary to understand the pathophysiological mechanisms and to provide better therapeutical approaches addressing tau pathology. Here, we characterized the contribution of CK1δ to AD-associated tau phosphorylation sites *in vitro* by using different techniques including MS, *in vitro* kinase assays and two-dimensional phosphopeptide analysis. The results were further supported by a cell-based assay and Western blot analysis using phospho-specific antibodies. Additionally, we demonstrated that CK1δ co-localized with tau in neuronal cells and that CK1δ-mediated phosphorylation led to an increased *in vitro* tau aggregation. With our data, we provide a comprehensive analysis of CK1δ-mediated site-specific tau phosphorylation and confirm a functional influence of CK1δ on tau aggregation.

## Materials and Methods

### Plasmid Constructs for Protein Expression

**FIGURE 1:**
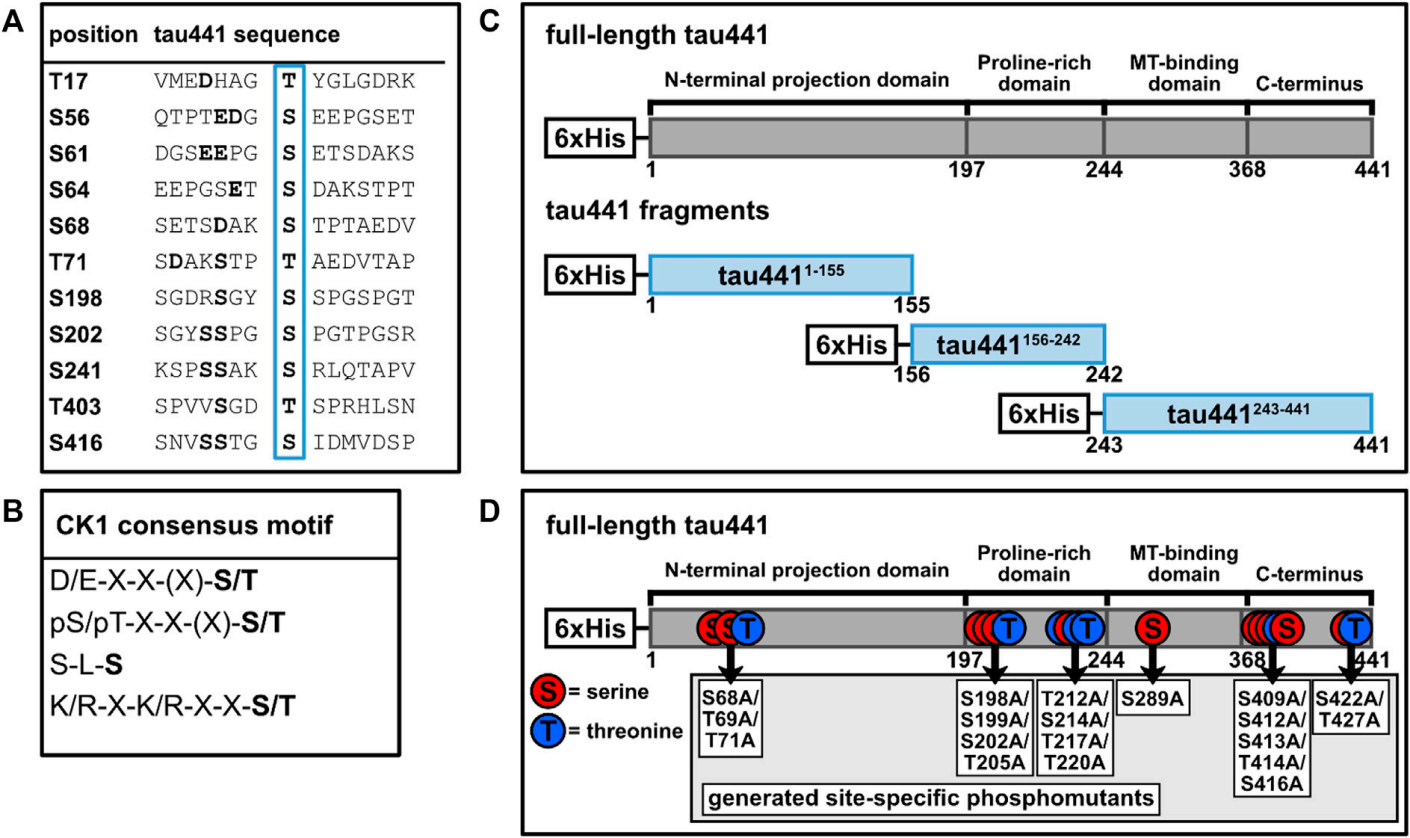
Tau441 contains several potential target sites for CK1. **(A)** Adjacent amino acid sequences of potential phosphorylation sites for CK1 on tau441 predicted by ScanSite 4.0 at low stringency according to Krismer et al. (2021). **(B)** Consensus motifs for CK1. **(C)** Tau441 fragments were generated according to different tau domains. **(D)** Tau441 phosphomutants were generated according to the positions of the predicted CK1 phosphorylation sites or the detection via MS analysis. MT: microtubule, pS/pT: pre-phosphorylated serine or threonine, S: serine, T: threonine, X: no amino acid preference.

The codon-optimized bacterial expression vector pET28a(+)tau441 encoding for N-terminal 6xHis-tagged human microtubule-associated protein tau (MAPT) isoform 4 (tau441) was synthesized by Biomatik (Kitchener, ON, Canada). Plasmids encoding for tau441 fragments (tau441^1-155^ and tau441^243-441^) and phosphorylation site mutants of full-length tau441 (tau441^S68A+T69A+T71A^, tau441^S198A+S199A+S202A+T205A^, tau441^T212A+S214A+T217A+T220A^, tau441^S289A^, tau441^S409A+S412A+S413A+T414A+S416A^ and tau441^S422A+T427A^) were created by using inverse PCR and the PCR primer pairs as indicated in Supplementary Table S1 (see Figure 1). Subsequently, PCR products were ligated. To generate plasmid pET28a(+)tau441^156-242^, Gibson Assembly^®^ was performed according to manufacturer’s instructions (New England Biolabs Inc., Ipswich, NY, United States). Sanger DNA sequencing (Eurofins Genomics, Munich, Germany) confirmed successful introduction of mutations.

### Expression and Purification of Recombinant 6xHis- and GST-Tagged Fusion Proteins

Plasmids encoding for 6xHis-tagged tau441 fragments or phosphorylation site mutants of full-length tau441 were transformed into *E. coli* SHuffle^®^ T7 Express (New England Biolabs Inc., Ipswich, NY, United States). Protein production was conducted in 450 ml lysogeny broth (LB) medium supplemented with 15 μg/ml kanamycin and induced with 0.5 mM IPTG at an OD_600_ of 0.6 AU for 3 h at 30°C. Bacteria were harvested by centrifugation for 10 min at 3,200 g and 4°C. Bacteria were lysed in lysis buffer composed of 50 mM sodium phosphate buffer (pH 7.0), 350 mM NaCl, 15 mM imidazole, 0.5% NP-40, 10% glycerol, 1 mM benzamidine, 0.25 g/L aprotinin and 10 mg lysozyme. Cell lysates were cleared by centrifugation for 20 min at 15,500 g and 4°C. According to Barghorn et al. (Barghorn et al., 2005), cleared lysates were boiled for 10 min and centrifuged for 30 min at 25,000 rpm and 4°C. Supernatant was mixed with 600 µl TALON^®^ Metal Affinity Resin (Takara Bio Inc., Kyoto, Japan) (50% (v/v) in PBS) and incubated at 4°C rotating overnight. Bound proteins were washed (50 mM sodium phosphate buffer (pH 7.0), 350 mM NaCl, 15 mM imidazole, 10% glycerol, 0.25 g/L aprotinin) and eluted stepwise (50 mM Na_2_PO_4_ pH 7.0, 350 mM NaCl, 350 mM imidazole, 10% glycerol, 0.25 g/L aprotinin) followed by dialysis using PD-10 desalting columns (Cytiva, Freiburg, Germany). Production of GST-tagged and 6xHis-tagged kinases was carried out as described previously (Knippschild et al., 1997; Roth et al., 2021b). All protein solutions were adjusted to 10% glycerol, quick frozen and stored at −80°C for subsequent use.

### *In Vitro* Kinase Assay and Two-Dimensional Phosphopeptide Analysis

The reaction was performed in a total volume of 15 µl containing the kinase buffer (25 mM Tris pH 7.5, 10 mM MgCl_2_, 0.1 mM EDTA, 10 µM ATP), 2 µCi [γ-^32^P]-ATP (only for radiometric determination), 300 nM CK1δ and 1 µg (4 µg for phosphopeptide analysis) substrate. As substrates either tau441, tau441 fragments or tau441 phosphorylation site mutants were used. Reaction was carried out for 30 min at 30°C in triplicates. Proteins were separated by SDS-PAGE on 10% gels followed by Coomassie blue staining. Radioactively labeled substrate bands were visualized on dried gels by autoradiography. For quantification of phosphorylated products, radioactively labeled substrate bands were excised from dried gels and phosphate incorporation was determined by Cherenkov counting (LC6000IC, Beckman Coulter, USA). *In vitro* phosphorylated tau441 (wild type), fragments and phosphorylation site mutants were analyzed by two-dimensional phosphopeptide analysis using standard protocols described previously (van der Geer and Hunter, 1994). *In vitro* phosphorylated proteins were separated via SDS-PAGE and transferred onto a PVDF membrane (Cytiva, Freiburg, Germany). Protein bands of interest were incubated with 5% (w/v) polyvinylpyrrolidone (in 10 mM acetic acid) at 37°C for 30 min, washed with 50 mM ammonium bicarbonate buffer and digested with 10 µg TPCK-trypsin. Digested proteins were further oxidized with performic acid on ice for 2 h. Radioactively labeled phosphopeptides were separated on cellulose TLC plates (Merck Millipore, Darmstadt, Germany) by electrophoresis at pH 1.9 (containing 6% (v/v) formic acid, 1.25% (v/v) acetic acid and 0.25% (v/v) pyridine in dH_2_O) followed by ascending chromatographic separation in buffer (composed of 37.5% (v/v) n-butanol, 7.5% (v/v) acetic acid and 25% (v/v) pyridine in dH_2_O). Radioactively labeled phosphopeptides were visualized by autoradiography.

### LC-MS/MS Analysis of Purified tau441

*In vitro* phosphorylated proteins were separated by SDS-PAGE and SDS gel pieces were in-gel digested with trypsin as described previously (Borchert et al., 2010). Extracted peptides were desalted using C18 StageTips (Rappsilber et al., 2007) and subjected to LC-MS/MS analysis. LC-MS/MS analyses were performed on an Easy-nLC 1200 UHPLC (Thermo Fisher Scientific Inc., Waltham, MA, United States) coupled to an QExactive HF Orbitrap mass spectrometer (Thermo Fisher Scientific Inc., Waltham, MA, United States) as described elsewhere (Schmitt et al., 2019). Peptides were eluted with a segmented gradient at a flow rate of 200 nl/min for 60 min, selecting seven most intensive peaks for fragmentation with HCD. The MS data was processed with MaxQuant software suite v.1.6.7.0 (Cox and Mann, 2008). Search for variable modification phosphorylation (STY) was enabled. Database search was provided against human (96817 entries) UniProt database using the Andromeda search engine (Cox et al., 2011). Since the goal of the analysis was to identify tau phosphorylation sites targeted by CK1δ, no global normalization of phosphorylation sites to proteome was performed. The amount of the identified phosphorylation sites within the aa 1 to 155, 156 to 242 and 243 to 441 were normalized to the total amount of the detected phosphorylation sites.

### Tau Aggregation and Thioflavin S Assay

Tau441 was phosphorylated *in vitro* as described above with 1,000 µM ATP and 300 nM GST-CK1δ. The reactions were incubated for 30 min at 30°C and afterwards centrifuged for 10 min at full speed and 4°C. Formation of cross-β structures of phosphorylated and non-phosphorylated tau441 (4 µM) in 100 mM Tris/HCl (pH 6.8) was induced with freshly prepared 150 µM arachidonic acid (10 mM in ethanol) as described previously (Barghorn et al., 2005; Chirita et al., 2005). Tau aggregation was detected by the addition of Thioflavin S (ThS). Changes in the emission fluorescence spectra with the excitation wavelength set at 430 nm and the emission wavelength set at 480 nm were monitored using a TriStar^2^ LB 942 multimode plate reader (Berthold Technologies, Bad Wildbad, Germany) at intervals of 1.5 min within 30 min. Data were displayed and fit to one-phase association exponential model using Prism 8 (GraphPad, San Diego, CA, United States).

### Cell Culture, Stable Transfection, and Treatment

Immortalized human neural progenitor cells (hNPCs) (ReNcell^®^ VM from Merck Millipore, Darmstadt, Germany) were expanded and maintained in proliferation medium (DMEM/F12 (Gibco/Life Technologies, Carlsbad, CA, United States) supplemented with 2% (v/v) B-27 neural supplement (Gibco/Life Technologies, Carlsbad, CA, United States), 2 μg/μl heparin (Stemcell Technologies Inc., Vancouver, BC, Canada), 20 ng/ml human basic fibroblast growth factor (bFGF) (Reprocell Inc., Glasgow, UK), 20 ng/ml human epidermal growth factor (EGF) (Sigma Aldrich, St. Louis, MO, United States), and 100 U/ml penicillin-streptomycin solution (Gibco/Life Technologies, Carlsbad, CA, United States)) as described previously (Roth et al., 2021a). For co-localization experiments, 0.3 × 10^6^ naïve hNPCs were seeded onto Matrigel-coated glass slides in a 6-well with differentiation medium (proliferation medium without growth factors) and differentiated for 2 weeks.

To generate a cell culture system for modeling the AD pathology, hNPCs were stably transfected with lentiviral DNA constructs pCSCW-APPSL-IRES-GFP and pCSCW-PSEN1(ΔE9)-IRES-mCherry, which encode full-length human APP695 with K670N/M671L/V717I (Swedish and London mutation) and GFP or human presenilin 1 with a deletion in exon 9 (PSEN1 (ΔE9)) and mCherry. The lentiviral DNA constructs were kindly provided by Prof. Dr. Doo Kim (Massachusetts General Hospital, Harvard Medical School, Charlestown, MA, United States). Lentiviral transduction and the subsequent enrichment of high-expressing transduced hNPCs via fluorescence-activated cell sorting (FACS) was performed according to Choi et al. (2014) and Kim et al. (2015). For the analysis of CK1δ-specific tau phosphorylation, 0.9 × 10^6^ transduced hNPCs were seeded into a Matrigel-coated 6-well and differentiated for 2 weeks. After 2 weeks, cells were treated with 1 µM PF-670462 (Sigma Aldrich, St. Louis, MO, United States) or DMSO as vehicle control for 24 h and subsequently lysed.

### Cell Lysis and Western Blot Analysis

Transduced hNPCs were washed with 20 mM Tris-HCl (pH 7.6) buffer containing 140 mM NaCl. Cell lysates used for Western blot analyses were prepared in RIPA lysis buffer composed of 50 mM Tris-HCl (pH 7.4), 150 mM NaCl, 0.5% sodium deoxycholate, 1% NP-40, 1 mM EDTA, 2 mM PMSF, 2 mM PNT, 1 mM Na_3_VO_4_, 1 mM NaF containing fresh phosphatase inhibitor cocktail (phosSTOP^TM^, Roche, Basel, Switzerland) and cOmplete^TM^ protease inhibitor cocktail (Roche, Basel, Switzerland). Cell lysates were cleared by centrifugation at 10,000 x g for 10 min at 4°C. 10 µg of cleared lysates were separated on 10% (v/v) gels in SDS-PAGE and transferred to a 0.2 µm PVDF membrane (Hybond-P, Amersham, Buckinghamshire, United Kingdom). Membranes were blocked in 5% (w/v) BSA in TBST for 1 h and incubated with anti-tau antibody (HT7, Thermo Fisher Scientific Inc., Waltham, MA, United States; 1:1000), anti-pSer202/pThr205-tau antibody (AT8, Thermo Fisher Scientific Inc., Waltham, MA, United States; 1:1000), anti-pSer214-tau antibody (D1Q2X, Cell Signaling Technology, Danvers, MA, United States; 1:1000), anti-pSer416-tau antibody (D7U2P, Cell Signaling Technology, Danvers, MA, United States, 1:1000) and anti-β-actin antibody (AC-15, Sigma Aldrich, St. Louis, MO, United States, 1:5000) overnight. Immunocomplexes were detected using a secondary antibody (horseradish-peroxidase (HRP)-conjugated anti-mouse or anti-rabbit antibody, 1:10,000). Immunoreactivity was detected by enhanced chemiluminescence using the Fusion FX imaging system (Vilber, Collégien, France). Signal intensities were quantitatively determined by using ImageJ (Schneider et al., 2012).

### Immunofluorescence Staining and Co-Localization Analysis

After differentiation and inhibitor treatment, cells were washed briefly with 1x PBS and fixed with 4% (v/v) paraformaldehyde in 1x PEM buffer (80 mM PIPES (pH 6.8), 1 mM EGTA, 5 mM MgCl_2_) at 4°C for 20 min followed by permeabilization with 0.3% (v/v) Triton-X 100 in 1x PEM buffer at RT for 5 min. Cells were washed briefly with 1x PEM and blocked with 5% (w/v) BSA in 1x PEM at RT for 30 min. Thereafter, cells were incubated with anti-tau antibody (HT7, Thermo Fisher Scientific Inc., Waltham, MA, United States; 1:500) at 4°C overnight. Cells were washed three times with 1x PEM and subsequently incubated with Alexa Fluor 488 anti-mouse antibody (Invitrogen^TM^, Carlsbad, CA, United States; 1:250) at RT for 1 h. Then, cells were washed three times with 1x PEM. Cultures were blocked again in 5% (w/v) BSA in 1x PEM at RT for 30 min, washed three times with 1x PEM and stained with anti-CK1δ antibody (ab10877, abcam, Cambridge, UK; 1:500) or anti-MAP2 antibody (Poly18406, BioLegend, San Diego, CA, United States; 1:500). After washing the 2D grown cells, they were incubated with Alexa Fluor 647 anti-goat antibody (Invitrogen^TM^, Carlsbad, CA, United States; 1:250) or Alexa Fluor 633 anti-rabbit antibody (Invitrogen^TM^, Carlsbad, CA, United States; 1:250) at RT for 1 h. Then, cells were washed three times with 1x PEM and stained with 0.1 μg/ml DAPI (Sigma Aldrich, St. Louis, MO, United States) at RT for 5 min. Washed cells were finally mounted with ProLong^TM^ Glass Antifade Mountant (Invitrogen^TM^, Carlsbad, CA, United States). Images were captured using the Leica SP8 confocal microscope (Leica Mikrosysteme Vertrieb GmbH, Wetzlar, Germany) at ×63 magnification.

Co-localization analysis was carried out using R (version 4.2.0, R Foundation for Statistical Computing, Vienna, Austria) and RStudio (version 2022.02.2, RStudio PBC, Boston, MA, United States). The required packages include imager (Barthelmé and Tschumperlé, 2019) and colocr (Ahmed et al., 2019). In brief, at least three regions of interest (ROI) were selected in the gray-scale image. The threshold value was set to 95. For evaluation of the co-localization the Pearson’s correlation coefficient (PCC) and the Mander’s overlap coefficient (MOC) were determined according to Ahmed et al. (2019). PCC describes the co-variance of the pixel intensities from both channels (Cy5, FITC). MOC describes the fraction of pixels from each channel (Cy5, FITC) with values above the background.

### Statistical Analysis

Results are presented as the mean of experiments at least performed in triplicates. Evaluation and statistical analysis of the results were performed using Prism 8 (GraphPad, San Diego, CA, United States). Statistical significance was tested by using the nonparametric Mann-Whitney U test. *p* values ≤0.05 (shown as * for *p* ≤ 0.05 and ns for not significant) were considered to be statistically significant.

## Results

### Tau441 Is Phosphorylated by CK1δ *in vitro*

Hyperphosphorylation of tau by various kinases plays an important role in the pathogenesis of AD. Previously, several studies demonstrated a potential role of CK1 in the hyperphosphorylation of tau that could be linked to the development of AD. The present study intends to demonstrate a specific involvement of CK1 in tau hyperphosphorylation and aggregation.

**FIGURE 2:**
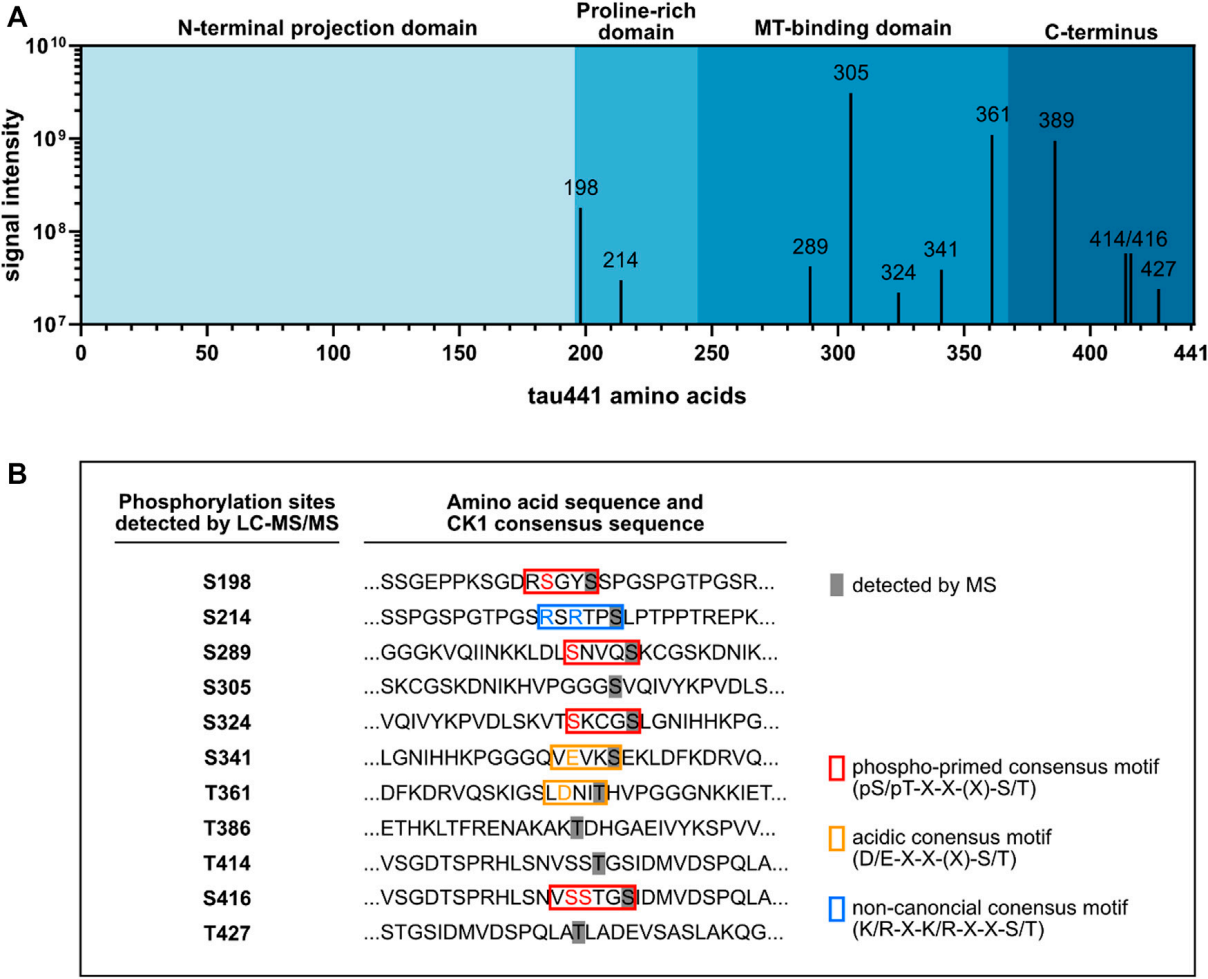
LC-MS/MS analysis identified ten tau441 phosphorylation sites targeted by CK1δ. Tau441 was recombinantly produced and used for *in vitro* phosphorylation by CK1δ. Phosphorylated tau441 was digested with trypsin and analyzed by LC-MS/MS. **(A)** Results of the LC-MS/MS analysis identified ten tau441 phosphorylation sites targeted by CK1δ mainly located in the MT-binding domain and the C-terminus of tau441. **(B)** Sequences of tau including the detected phosphorylation sites. Detected phosphorylation sites are covered in grey. Phospho-primed, acidic or non-canonical consensus motifs of CK1 are marked in red, orange and blue, respectively.

According to the canonical consensus sequence for CK1 substrates, the longest tau isoform (tau441) contains several putative target sites for CK1-mediated phosphorylation (Figure 1A). Initially, mass spectrometric examination of CK1δ-phosphorylated tau441 was used to detect whether CK1δ is able to phosphorylate tau441. Therefore, full-length human N-terminal 6xHis-tagged tau441 encoded on pET28a(+) (purchased from Biomatik (Kitchener, ON, Canada)) was recombinantly expressed in *E. coli*, purified via immobilized metal affinity chromatography and subsequently phosphorylated by human recombinant CK1δ *in vitro*. LC-MS/MS data generated from *in vitro* phosphorylated and trypsin-digested tau441 revealed 91.8% and 94.1% sequence coverage of non-phosphorylated tau441 and CK1δ-phosphorylated tau441, respectively, based on the amino acid sequence of the longest human tau isoform tau441 (UniProt: P10636-8). Additionally, MS analysis did not detect any phosphorylation of non-phosphorylated tau441. In total, we detected phosphopeptides corresponding to ten different phosphorylation sites (Figure 2A; Table 1). In the majority of cases, precise phosphorylation sites could be identified. A clear identification was not possible for the residues Thr414 and Ser416, which belong to a phosphopeptide sequence containing four closely spaced potential CK1-specific phosphorylation sites (Ser412, Ser413, Thr414 and Ser416).

**TABLE 1:** LC-MS/MS analysis of tau441 *in vitro* phosphorylated by CK1δ. Tau441 was recombinantly expressed in *E. coli* and purified. Subsequently, tau441 was phosphorylated by CK1δ *in vitro*, digested with trypsin and analyzed by LC-MS/MS. Positions of the identified phosphorylation sites within tau441 (indicated with **p**) as well as phosphorylation probability data are shown.

Site position	Probability [%]	Modified sequence	CK1δ intensity	AD association
S198	100	SGY**p**SSPGSPGTPGSR	1.8*10^8^	yes (Morishima-Kawashima et al., 1995b; Hanger et al., 1998)
S214	100	TP**p**SLPTPPTREPK	3.0*10^7^	yes (Hanger et al., 1998; Kinoshita et al., 2006)
S289	100	KLDLSNVQ**p**SK	4.2*10^7^	yes (Hanger et al., 2007)
S305	100	HVPGGG**p**SVQIVYKPVDLSK	3.1*10^9^	no
S324	100	CG**p**SLGNIHHKPGGGQVEVK	2.2*10^7^	no
S341	100	**P**SEKLDFKDR	3.9*10^7^	no
T361	100	IGSLDNI**p**THVPGGGNK	1.1*10^9^	no
T386	100	AK**p**TDHGAEIVYK	9.5*10^8^	no
S416/T414	60.5/34.3	HLSNVSS**p**TG**p**SIDMVDSPQLATLADEVSASLAK	5.8*10^7^	yes/yes (Hanger et al., 2007)
T427	67.7	HLSNVSSTGSIDMVDSPQLA**p**TLADEVSASLAK	2.4*10^7^	yes (Hanger et al., 2007)

Most of the phosphorylation sites, which could be detected in our study, were recently identified by Hanger et al. (2007). Additionally, we found two phosphorylation sites, Ser324 and Thr427, that have not been identified so far. However, we did not detect CK1δ-mediated phosphorylation of Thr17, Ser46/Thr50, Thr95, Thr101/Thr102, Ser113, Ser131, Thr149, Thr169, Ser184, Ser208, Ser210/Thr212, Ser237/Ser238, Ser241, Ser258, Ser262, Thr263, Ser285, Ser341, Ser352/Ser356/Thr361, Thr373, Ser412/Ser413, Ser433, and Ser435, which were recently identified by using LC-MS/MS (Hanger et al., 2007). Of the ten phosphorylation sites, which we detected by LC-MS/MS analysis, five were located within the MT-binding domain, which plays a special role in the interaction of tau with the microtubule (Lee et al., 1989) (Figure 2A). CK1-specific consensus sequences were identified for Ser198, Ser214, Ser289, Ser324, Thr361 and Ser416 (Figure 2B). No typical CK1-specific consensus sequence was observed for Ser305, Thr386, Thr414 and Thr427. Interestingly, five out of ten CK1δ-specific phosphorylation sites (including Ser198, Ser214, Ser289, Thr414/Ser416 and Thr427) have been associated to AD in previous studies (see Table 1).

**FIGURE 3:**
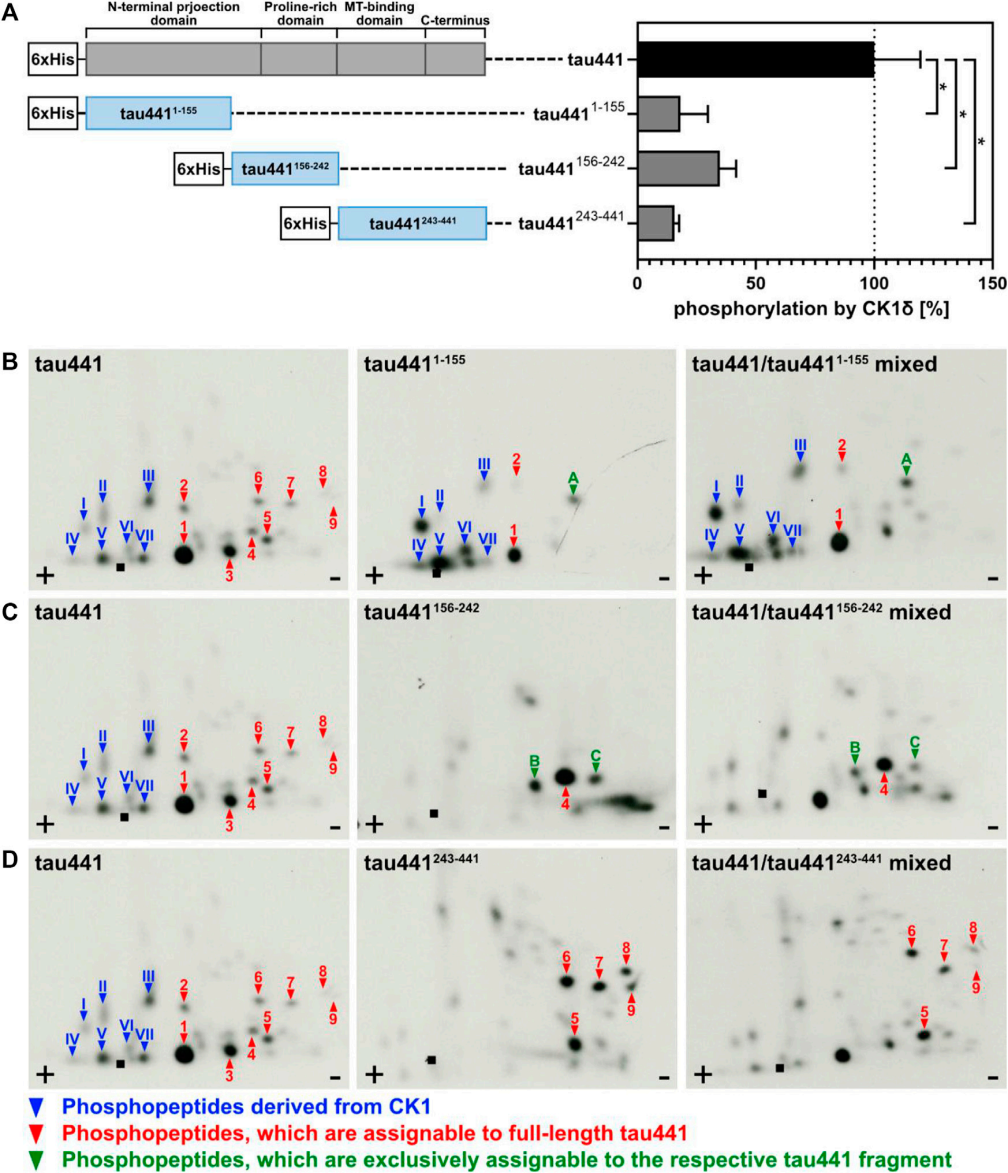
Tau441 and tau441 fragments are phosphorylated by CK1δ *in vitro*. Tau441 as well as tau441^1-155^, tau441^156-242^ and tau441^243-441^ fragments were phosphorylated by CK1δ *in vitro*. **(A)** After separation of phosphorylated proteins by SDS-PAGE followed by Coomassie staining, determination of phosphate incorporation into the different tau proteins was performed by Cerenkov counting. Data is shown as mean values of the experiments performed in triplicates and error bars represent the standard deviation. Data was normalized to full-length tau441. Statistical significance was tested using the nonparametric Mann-Whitney U test. * indicates *p* ≤ 0.05. MT: microtubule. Full-length tau441 as well as **(B)** tau441^1-155^, **(C)** tau441^156-242^ and **(D)** tau441^243-441^ were phosphorylated by CK1δ *in vitro*, processed and analyzed by two-dimensional phosphopeptide analysis. Arabic numerals indicate major phosphorylated peptides, which can be assigned to full-length tau441. Phosphopeptides, that are exclusively phosphorylated in the respective tau441 fragment are indicated with letters. Roman numerals indicated phosphopeptides that correspond to autophosphorylated CK1δ. ■: loading point of samples, +: anode, −: cathode.

**FIGURE 4:**
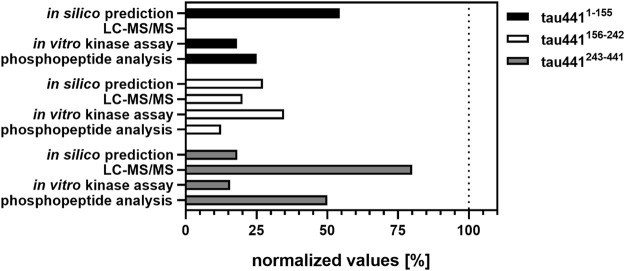
Overview of the results obtained from *in silico* prediction, LC-MS/MS analysis, *in vitro* kinase assays and phosphopeptide analysis. Amount of the identified phosphorylation sites (*in silico* prediction of CK1-specific phosphorylation sites by ScanSite 4.0 at low stringency according to Krismer et al. (2021) and LC-MS/MS analysis) or phosphopeptides (visualized by phosphopeptide analysis) were normalized to the total amount of phosphorylation sites or phosphopeptides detected by the respective method. Data of the *in vitro* kinase assays is shown as mean values of the experiments performed in triplicates and was normalized to full-length tau441.

In order to support the results obtained by MS analysis, fragments of tau441 were generated. Therefore, the sequence of tau441 was divided into three shorter fragments (tau441^1-155^, tau441^156-242^ and tau441^243-441^). Each of them contains a specific domain of tau441 (Figure 3). Phosphorylation by CK1δ has been observed for all fragments, indicating that phosphorylation sites for CK1δ are located within all domains of tau441 protein (Figure 3). Most intense phosphorylation could be observed for fragment tau441^156-242^, which contains the second most predicted CK1-specific phosphorylation sites on tau441 (Figure 4). MS analysis revealed that most of the phosphorylation sites are located on tau441^243-441^, which cannot be supported by the findings obtained from the *in vitro* kinase assay measuring the transfer of radioactively labeled phosphate.

After MS analysis and *in vitro* kinase assays, two-dimensional phosphopeptide analyses were performed to confirm the results obtained from the previous experiments. Due to technical limitations, it was not possible to fully separate the protein band of tau441 from that of CK1δ by SDS-PAGE. However, by performing phosphopeptide analysis also with CK1δ alone, phosphopeptides corresponding to the autophosphorylated kinase could be clearly identified (see Supplementary Figure S1, roman numerals). Two major phosphopeptides and one additional phosphopeptide, which cannot be allocated to full-length tau441, were visible in the autoradiograph of tau441^1-155^ (peptides 1, 2 and A in Figure 3B). The autoradiograph of tau441^156-242^ showed only one major full-length tau441-associated phosphopeptide and two additional phosphopeptides, which are exclusively phosphorylated in the fragment (peptides 4, B and C in Figure 3C). The phosphorylation pattern of tau441^243-441^ showed most major peptide signals that could be assigned to full-length tau441 (peptides 5 to 9 in Figure 3D).

In sum, data obtained from MS analysis and classical biochemical approaches provide evidence that CK1δ is able to phosphorylate tau441 at several different phosphorylation sites. Interestingly, the distribution of *in silico* predicted CK1-specific phosphorylation sites on tau441 could be verified by *in vitro* kinase assays especially for tau441^156-242^ and tau441^243-441^ (Figure 4). In contrast to that, data obtained from MS analysis clearly supported the results of the phosphopeptide analysis. In conclusion, most CK1δ-specific phosphorylation sites on tau441 are located within its MT-binding and the C-terminal domain, both located on fragment tau441^243-441^. To prove this right, we performed *in vitro* kinase assays and phosphopeptide analysis with tau441 phosphomutants in combination with cell-based assays, which together provide enough information about specific phosphorylation sites within the domains.

### CK1δ Targets AD-Associated Phosphorylation Sites on Tau

**FIGURE 5:**
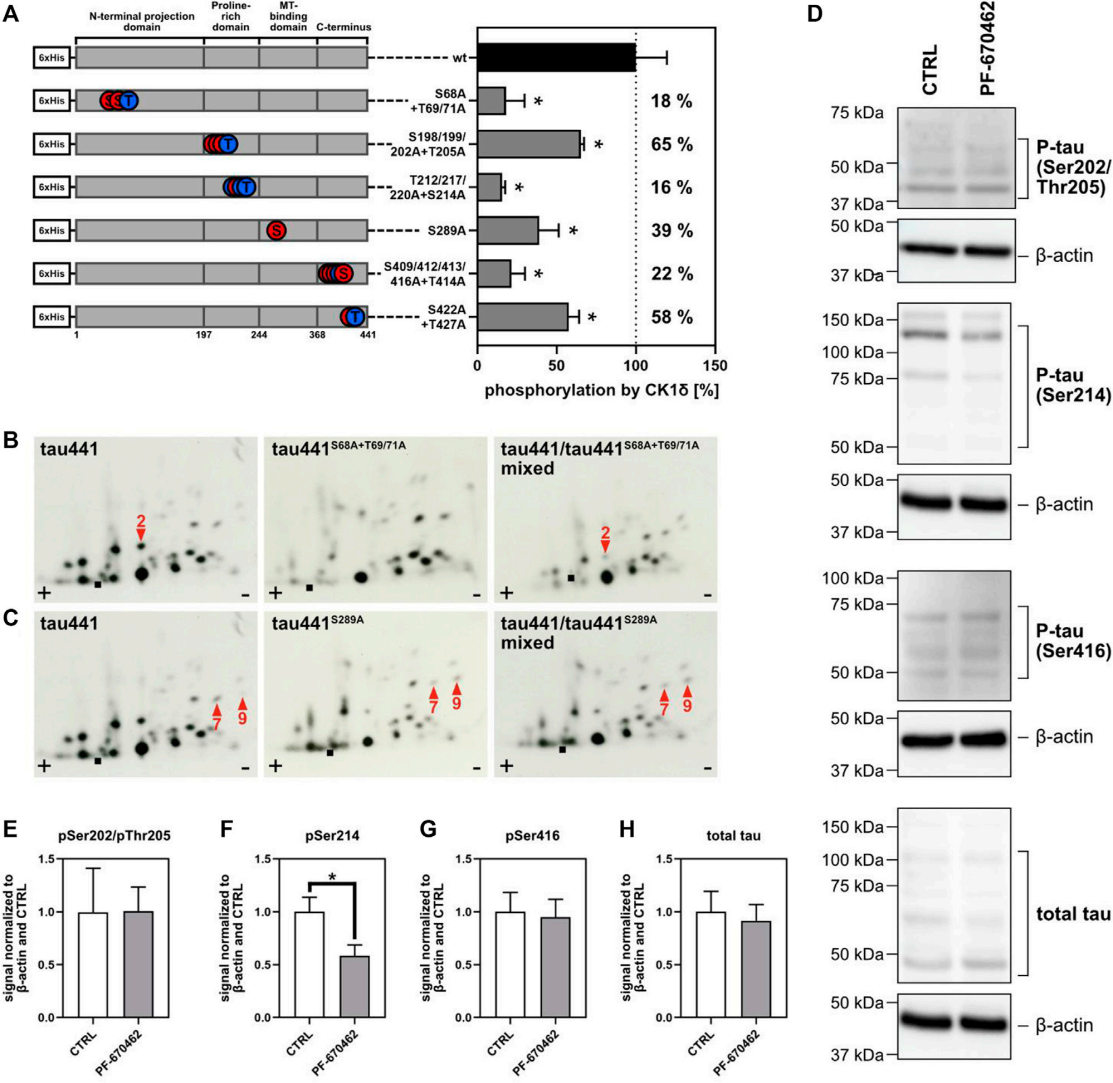
CK1δ targets AD-associated phosphorylation sites on tau441. **(A)** Tau441 and tau441 phosphomutants were phosphorylated by CK1δ *in vitro* using radioactively labeled ^32^P phosphate. Phosphate incorporation was quantified and normalized to wild type (wt) tau441. Information about the localization of the mutated phosphorylation sites on tau441 are shown on the left side of the figure. A: alanine, S: serine, T: threonine, wt: wild type. Phosphopeptide analysis of CK1δ-phosphorylated tau441 phosphomutants **(B)** tau441^S68A+T69A+T71A^ and **(C)** tau441^S289A^. Arrow position indicate the loss of a major phosphopeptide or changes in the phosphorylation pattern. Phosphopeptide analysis of mixed samples confirm the identity of the marked phosphopeptides. ■: loading point of samples, +: anode, −: cathode. **(D)** Transduced and differentiated hNPCs were treated with 1 µM PF-670462 or DMSO as vehicle control (CTRL). Tau phosphorylation was determined by Western blots and antibodies targeting pSer202/pThr205-tau, pSer214-tau, pSer416-tau and total tau. β-actin was used as loading control. **(E–H)** Western blot signal intensities were quantified and normalized to β-actin and CTRL (vehicle control). Experiments were performed in triplicates and are shown as mean values. Error bars represent the standard deviation. Statistical significance was tested using the nonparametric Mann-Whitney U test with *p ≤ 0.05.

To validate potential AD-associated phosphorylation sites predicted by the consensus motif via Scansite 4.0 or identified by MS analysis, six tau441 phosphomutants encompassing amino acids 68–71, 198–205, 212–220, 289, 409–416, and 422–427 were designed and generated by using primers given in Supplementary Table S1. Equal amounts of wild type tau441 and tau441 phosphomutants were then subjected to *in vitro* phosphorylation by CK1δ and phosphate incorporation was quantified. Mutation of Ser68/Thr69/Thr71, Thr212/Ser214/Thr217/Thr220, Ser289 as well as Ser409/Ser412/Ser413/Thr414/Ser416 showed major reduction of CK1δ-mediated phosphorylation by 18%, 16%, 39%, and 22% compared to wild type tau441 (see Figure 5A). Only minor reduction in phosphorylation was observed for Ser198/Ser199/Ser202/Thr205 and Ser422/Thr427 with 65 % and 58% compared to wild type tau441.

In addition, the significance of these results was further confirmed by phosphopeptide analysis using tau441 (wild type) and corresponding phosphomutants tau441^S68A+T69A+T71A^, tau441^S198A+S199A+S202A+T205A^, tau441^T212A+S214A+T217A+T220A^, tau441^S289A^, tau441^S409A+S412A+S413A+T414A+S416A^ and tau441^S422A+T427A^. As expected from *in silico* prediction (Figure 1A), one phosphopeptide (peptide 2) is missing in the phosphopeptide analysis of tau441^S68A+T69A+T71A^ compared to wild type tau441 (Figure 5B). Additionally, phosphopeptide analysis of tau441^S289A^ showed changes in the phosphorylation pattern concerning phosphopeptides 7 and 9, thereby confirming that Ser289 can by phosphorylated by CK1δ *in vitro* (Figure 5C). No new information has arisen from the phosphopeptide patterns of tau441^S198A+S199A+S202A+T205A^, tau441^T212A+S214A+T217A+T220A^, tau441^S409A+S412A+S413A+T414A+S416A^ and tau441^S422A+T427A^ (Supplementary Figure S2).

To determine whether CK1δ modulates tau phosphorylation in neurons, transduced hNPCs were differentiated for 2 weeks and treated with 1 µM PF-670462 or DMSO as vehicle control for 24 h. After cell lysis, site-specific tau phosphorylation was determined by Western blot and phospho-specific tau antibodies targeting pSer202/pThr205, pSer214 or pSer416. We observed that treatment with PF-670462 did not affect the expression of tau (Figure 5D). Additionally, tau phosphorylation in cells treated with PF-670462 was significantly decreased at Ser214 verifying the results obtained by MS analysis and *in vitro* kinase assay. However, CK1δ-specific inhibition by PF-670462 did not affect tau phosphorylation at Ser202/Thr205 and Ser416 (Figure 5E).

### CK1δ Co-Localizes With tau441 in Neuronal Cells

**FIGURE 6:**
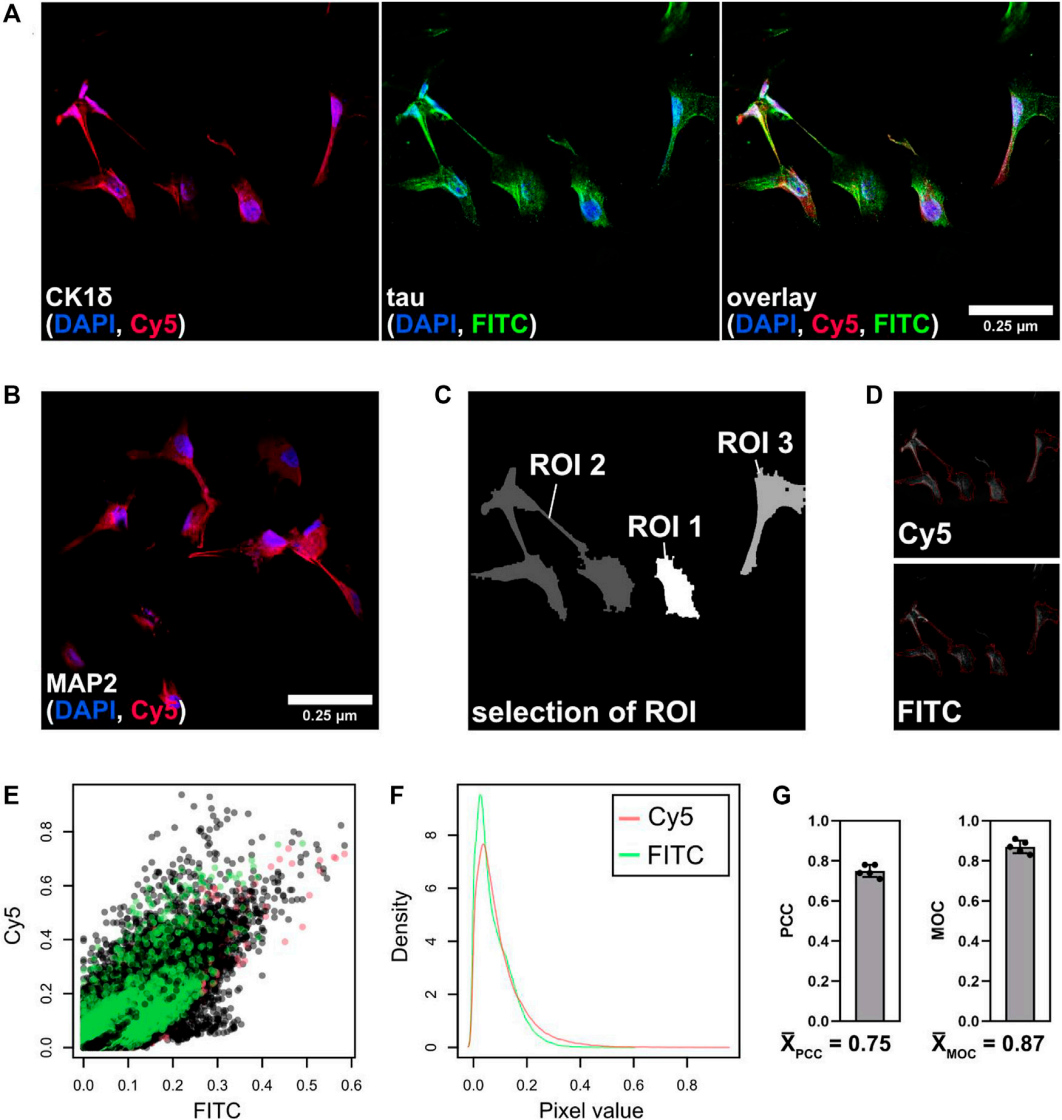
Co-localization of tau and CK1δ in neuronal cells. Immunofluorescence was performed with differentiated human neuronal cells. **(A)** Cells were fixed and stained with DAPI (blue, nuclei), anti-CK1δ goat antibody/Alexa Fluor 647 anti-goat antibody (Cy5/red, CK1δ) and anti-tau mouse antibody/DyLight 488 anti-mouse antibody (FITC/green, tau). **(B)** Differentiation level was examined by staining of neuron-specific marker MAP2. Images were taken at ×63 magnification using the Leica SP8 confocal microscope (Leica Mikrosysteme Vertrieb GmbH, Wetzlar, Germany). Scale bar: 0.25 µm. **(C,D)** Merged images were processed and analyzed using R according to Ahmed et al. (2019). **(C)** Low-resolution image (pixel set) of the merged image with selected ROIs. **(D)** Selected ROIs are highlighted (red line) within both channels (Cy5, FITC). **(E)** Raw pixel intensities of both channels (Cy5, FITC) from three selected regions (shown in black, green, and red). **(F)** Density of the pixel values of both channels (Cy5 (red) and FITC (green)) from summarized ROIs. **(G)** Analysis of PCC and MOC according to (Ahmed et al., 2019) (*n* = 5). Values are given as mean and error bars represent the standard deviation. MOC: Mander’s overlap coefficient, PCC: Pearson’s correlation coefficient, ROI: region of interest.

We further explored whether tau and CK1δ are co-locating in human neuronal cells that were generated from hNPCs. By double immunofluorescence staining and confocal microscopy, the expression of tau and CK1δ was visualized in neuronal cells (Figure 6A). Control staining with secondary antibodies only is depicted in Supplementary Figure S3. Neuronal differentiation level was verified by immunofluorescence staining of the neuron-specific marker MAP2 shown in Figure 6B. Analysis of PCC and MOC was performed to determine the subcellular co-localization of CK1δ with tau Figures 6C–G.

As shown in Figure 6A, immunofluorescence staining of tau as well as CK1δ revealed a variety of subcellular localizations. A clear staining pattern for both proteins was especially observed around the nuclei and in the cell body. Co-localization was determined by performing PCC and MOC analysis using R (Figures 6C–G). Co-localization analyses gave a PCC of 75% (0.75) and a MOC of 87% (0.87) for the overlay of CK1δ (Cy5) with tau (FITC) (Figure 6G) indicating a strong co-localization of CK1δ and tau in differentiated neuronal cells.

### CK1δ-Mediated Phosphorylation has an Impact on Tau Aggregation

Hyperphosphorylation of tau by various kinases leads to the aggregation of tau into PHFs causing NFT formation and neuronal cell death in AD. We therefore considered the possibility that CK1δ enhances tau aggregation by phosphorylating tau at AD-associated phosphorylation sites.

**FIGURE 7:**
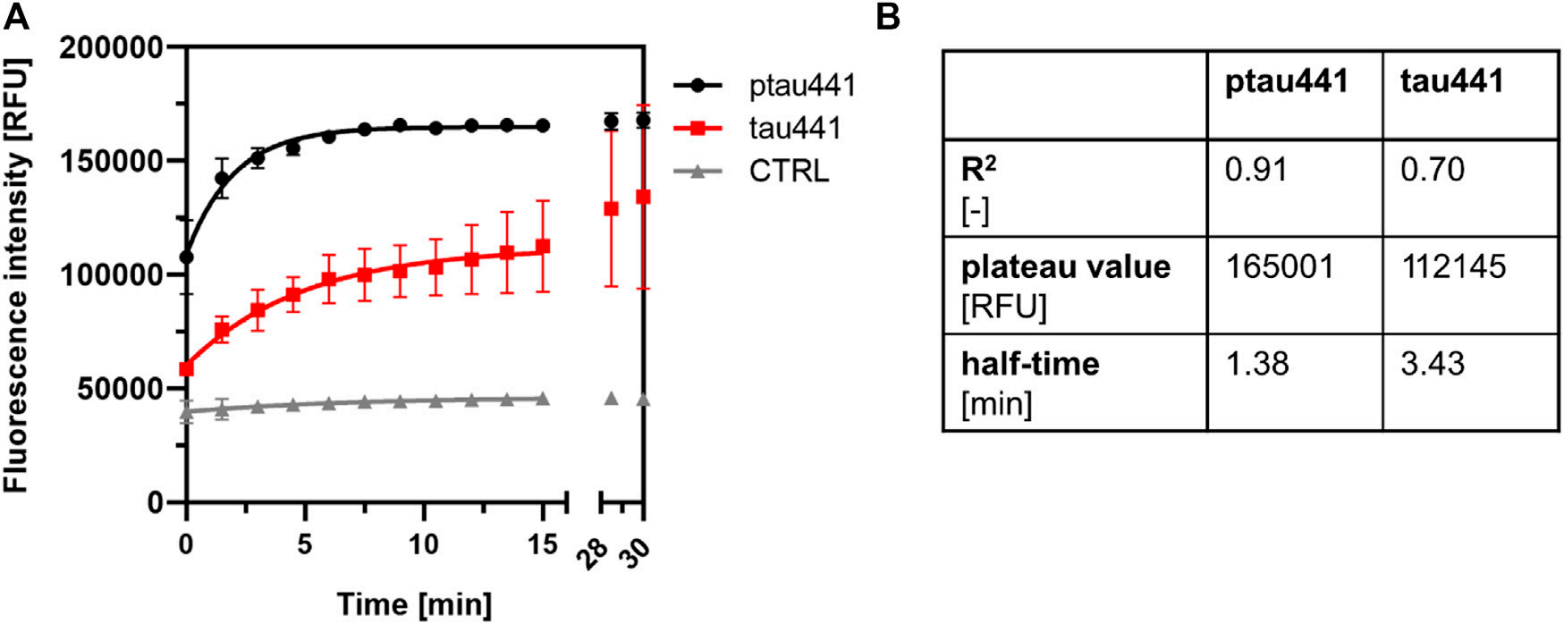
CK1δ-mediated phosphorylation is linked to tau aggregation. **(A)** Tau aggregation assay was performed with CK1δ-phosphorylated tau441 (ptau441) and non-phosphorylated tau441 (tau441). Control reaction (CTRL) was performed with standard reaction mix without protein. Tau aggregation was measured by the ThS assay at 480 nm. Shown are the mean values of the triplicates and the error bars of the standard deviation. Data were fit to one-phase association exponential model within the first 15 min. **(B)** Amount of formed aggregates and velocity of aggregation was calculated as plateau value [RFU] and half-time [min]. CTRL: control reaction, ptau441: CK1δ-phosphorylated tau441, RFU: relative fluorescence units, tau441: non-phosphorylated tau441.

Tau aggregation assay was performed with CK1δ-phosphorylated and non-phosphorylated tau441. Standard reaction mix without protein served as a negative control. The tau aggregation kinetics was strongly affected by the phosphorylation of CK1δ (Figure 7A). Phosphorylation by CK1δ highly increased the formation of tau441 aggregations (correlating with an increased plateau value) as well as the aggregation velocity (correlating with a decreased half-time of the aggregation kinetics) (Figure 7B).

## Discussion

Hyperphosphorylation of tau leads to the formation of PHF and can only be achieved by the activities of various kinases. A wide range of kinases have been found to phosphorylate tau and contribute to this pathophysiological hallmark of AD (Grundke-Iqbal et al., 1986; Kosik et al., 1986; Brion et al., 1991). Here, our data strongly support previous findings that CK1δ is one of the kinases specifically phosphorylating tau441 at several AD-associated residues. Furthermore, we show that CK1δ and tau are co-localized in neuronal cells and that CK1δ-mediated phosphorylation affects tau aggregation.

Initially, site-specific phosphorylation of tau441 was predicted for CK1 canonical consensus motifs using ScanSite 4.0 revealing ten potential phosphorylation sites. Complementary information was obtained via MS analysis resulting in ten potential CK1δ-specific phosphorylation sites. Classical biochemical methods (such as *in vitro* kinase assays and two-dimensional phosphopeptide analysis) revealed that CK1δ prefers to phosphorylate tau441 within the MT-binding and C-terminal domain (aa 244-441) as well as the proline-rich domain (aa 197-244), which is in line with the findings of several other studies, demonstrating that most of the known AD-associated phosphorylation sites are located within the central region (172-251) and the C-terminal region (aa 368-441) (Liu et al., 2007; Rudrabhatla et al., 2011; Noble et al., 2013).

**FIGURE 8:**
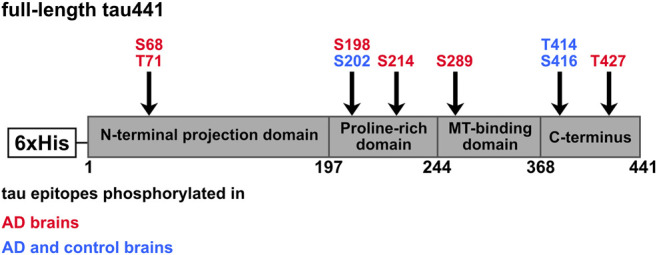
Potential CK1δ-specific phosphorylation sites on tau protein. Tau441 phosphorylation sites were predicted *in silico* or detected experimentally via MS analysis, *in vitro* kinase assay or phosphopeptide analysis. Shown are CK1-specific phosphorylation sites, which were predicted/identified with at least two methods. Red: Tau epitopes phosphorylated in AD brain. Blue: Tau epitopes phosphorylated in AD and control brains.

Combined results of *in silico* approaches and MS analysis showed that CK1δ specifically phosphorylates the AD-associated phosphorylation sites Ser68, Thr71, Ser198, Ser214, Ser289, Thr414/Ser416 and Thr427 (Figure 8; Table 2). Biochemical methods and Western blot analysis with phospho-specific antibodies of cell lysates obtained from treatment with CK1δ-specific inhibitor were used to verify these findings. Our results demonstrate that not all predicted sites, which are included in the CK1 consensus motif suggested by Krismer et al. (2021) are phosphorylated by CK1δ *in vitro*. Additionally, several CK1δ-targeted phosphorylation sites detected by MS analysis could not be confirmed in other *in vitro* experiments (Ser198, S202, Thr427) and vice versa. *In vitro* experiments based on *in vitro* kinase assays confirmed that the AD-associated phosphorylation sites Ser68 and/or Thr71 and Ser289 on tau441 are targeted by CK1δ. Although Ser68 and/or Thr71 were not detected by MS analysis, *in vitro* kinase assays and phosphopeptide analysis clearly identified these phosphorylation sites as CK1δ-specific highlighting the potential of these methods. The failure to detect these phosphorylation sites by MS analysis could have been due to difficulties in separation, the loss of phosphorylation at these residues during sample preparation or reduced peptide lengths (Liu et al., 2005; Vandermarliere et al., 2013; Solari et al., 2015). Differences between *in vitro* kinase assay and phosphopeptide analysis could be explained by technical limitations within sample preparation prior to phosphopeptide mapping. For sample preparation, we performed on-membrane digestion with trypsin, that has distinct advantages over in-gel digestion. However, we experienced a loss of some (phopho)peptides, that is probably caused by peptide precipitation and unpolymerized acrylamide cross-linked to proteins interfering tryptic digestion, that leads to inefficient tryptic peptide digestion and insufficient membrane detachment (Luque-Garcia and Neubert, 2009). Phosphorylation of Thr414/Ser416 was detected *in silico* and experimentally via MS analysis and *in vitro* kinase assays, but Ser416 could not be verified by Western blot analysis indicating no physiological relevance for the phosphorylation of Ser416 by CK1δ. However, significant physiological relevant phosphorylation by CK1δ was demonstrated for Ser214. Previous studies emphasize that the combination of results obtained from different methods is essential to reliably provide evidence for the phosphorylation of certain amino acid residues (Bischof et al., 2013; Dephoure et al., 2013; Ianes et al., 2016; Meng et al., 2016; Meng et al., 2019). Considering all methods, which were performed, it is very likely that Ser68/Thr71, Ser214 and Ser289 are targeted by CK1δ.

**TABLE 2:** AD-associated phosphorylation sites of CK1 within tau. Results were obtained from different methods including different sources of tau as indicated. Positive identified phosphorylation sites are shown as “✓” and positive results within a tau441-phosphomutant including multiple amino acid are shown as “✓*.”

	Ser68	Thr69	Thr71	Ser198	Ser199	Ser202	Thr205	Thr212	Ser214	Thr217	Thr220	Ser289	Thr403	Ser409	Ser412	Ser413	Thr414	Ser416	Ser422	Thr427
*in silico* prediction (tau441)	✓		✓	✓		✓							✓					✓		
MS analysis (recombinant tau441)				✓					✓			✓					✓	✓		✓
*in vitro* kinase assay (recombinant tau441)	✓*	✓* (minor)	✓*	✓	X	✓*	✓* (minor)													
phosphopeptide analysis (recombinant tau441)	✓*			✓	X															
Western blot (endogenous tau)	x	x	x	X	X			x	✓	x	x	x	x	x	x	x	x		x	x

✓: positive result, ✓*: positive result with a tau441-phosphomutant including multiple amino acid changes, x: not performed.

Ser68 and/or Thr71 are newly identified CK1δ-specific phosphorylation sites, which are tightly connected to the pathogenesis of AD. Previously, comprehensive MS analysis identified phosphorylated Ser68 and Thr71 in brain tissue isolated from patients with AD (Hanger et al., 2007). So far, only a few kinases have been identified to phosphorylate Thr69 or Thr71 including GSK3 (Hanger et al., 2009) or AMP-activated protein kinase (AMPK) (Thornton et al., 2011), respectively. Interestingly, *in vitro* phosphorylation of Thr71 was observed after combining GSK3β with CK1δ indicating a potential priming role of CK1δ for GSK3β (Hanger et al., 2007). Hyperphosphorylated tau in PHFs was reported to contain several double-site phosphorylation residues, such as Thr212/Ser214. Comparable to Ser68 and Thr71, phosphorylation of Thr212/Ser214 is absent in biopsy-derived normal tau (Matsuo et al., 1994). Phosphorylation of Ser214 is of particular interest, because its phosphorylation alone potentially leads to the disruption of microtubule binding and reduces the affinity of tau for microtubules (Illenberger et al., 1998). Phosphorylation of Ser214 was detected to be mediated by PKA (Zheng-Fischhöfer et al., 1998; Hanger et al., 2007), Akt (Ksiezak-Reding et al., 2003), CDK5, GSK3 and CK1δ (Hanger et al., 2007; Hanger et al., 2009). Among the identified CK1δ-specific phosphorylation sites, Ser289 is located within the microtubule-binding domain of tau important for its microtubule-binding property. Phosphorylation events within this domain were associated with conformational changes of tau and its disruption from microtubules (Biernat et al., 1993; Liu et al., 2007). Other kinases, which are involved in the phosphorylation of Ser289 are checkpoint kinase (Chk)1, Chk2 (Mendoza et al., 2013) and GSK3 (Hanger et al., 2009).

In addition, we provide evidence that tau co-localizes with endogenous CK1δ in differentiated neuronal cells, which was demonstrated in double immunofluorescence staining of both proteins. Co-localization is required for the interaction of both proteins and subsequent phosphorylation. These findings support the hypothesis that CK1δ has a possible role in the modulation of tau, which is further supported by the findings that CK1 is an active physiological kinase in neuronal cells and that the expression of CK1δ is strongly increased in AD brains probably leading to severe and pathological disruption of tau-MT binding (Ghoshal et al., 1999; Yasojima et al., 2000; Hanger et al., 2007).

CK1δ phosphorylates tau at specific AD-associated residues *in vitro* and in cells, it co-localizes with tau in neuronal cells and its expression is up-regulated in the brain of patients suffering from AD (Ghoshal et al., 1999). To directly demonstrate the functional effect of CK1δ-mediated phosphorylation on tau aggregation, we performed *in vitro* tau aggregation assays with pre-phosphorylated tau441 and non-phosphorylated tau441. Tau aggregation assay is not only used to study mechanisms of tau misfolding and aggregation, but it is also a robust tool for the screening of drugs, that interfere and inhibit tau aggregation. The relevance of this assay was previously demonstrated by Pickhardt et al. (2005). In this study, anthraquinone-based small molecule inhibitors were identified in an initial drug screening for their ability to prevent tau aggregation by using the tau aggregation assay. Promising compounds were further successfully identified for their ability to prevent tau aggregation in a cell-based assay using a genetically modified neuroblastoma (N2a) cell line. By using the tau aggregation assay in our experimental set-up, a remarkable increase in the aggregation kinetics of pre-phosphorylated tau441 could be observed. In aggregated PHF-tau, several amino acid residues were shown to be phosphorylated including Ser68, Ser69, Thr71, Ser184, Ser185, Ser202, Thr205, Ser208, Ser210, Thr212, Ser214, Thr231, Ser235, Ser258, Ser262, Ser289, Thr403, Ser412, Thr414/Ser416, Ser422, Thr427, Ser433 and Ser435 (Wolozin et al., 1986; Morishima-Kawashima et al., 1995a; Zheng-Fischhöfer et al., 1998; Hanger et al., 2007; Despres et al., 2017). Within this study, several phosphorylation sites, which are associated to tau aggregation, were predicted to be phosphorylated by CK1 *in silico* (Ser68, Thr71, Ser198, Ser202, Thr403, Ser416) or experimentally identified (Ser68, Thr71, Ser214, Ser289). Results obtained from our aggregation experiments demonstrated a potential role of CK1δ in tau aggregation, which has already been shown for several other kinases including TTBK1 and Fyn (Xu et al., 2010; Briner et al., 2020). In a bi-transgenic mouse model (overexpressing TTBK1 and P301L tau mutant) enhanced tau phosphorylation at Ser202/Thr205, Ser262/Ser356, Ser396/Ser404, Ser422 was observed leading to increased accumulation of tau aggregates (Xu et al., 2010). TTBK1 is known to be a neuron-specific kinase that regulates tau phosphorylation. Interestingly, TTBK1 shares high homology and characteristics with CK1δ explaining why both kinases are assigned to the CK1 family within the phylogenetic kinase tree (Liachko et al., 2014).

In summary, we clearly identified AD-associated tau phosphorylation sites, which can be targeted by CK1δ. Furthermore, we demonstrated that CK1δ co-localizes with tau in differentiated neuronal cells. For the first time, we provide experimental proof that CK1δ-mediated phosphorylation plays an important role at least in the *in vitro* aggregation of tau. Therefore, our findings clearly support the assumption that CK1δ has an essential role in tau hyperphosphorylation and the pathogenesis of AD.

## Data Availability

The original contributions presented in the study are included in the article/Supplementary Material, further inquiries can be directed to the corresponding author.

## References

[B1] Ahmed M. Lai T. H. Kim D. R. (2019). Colocr: an R Package for Conducting Co-localization Analysis on Fluorescence Microscopy Images. PeerJ 7, e7255. 10.7717/peerj.7255 31309005 PMC6612416

[B2] Anand R. Gill K. D. Mahdi A. A. (2014). Therapeutics of Alzheimer's Disease: Past, Present and Future. Neuropharmacology 76, 27–50. 10.1016/j.neuropharm.2013.07.004 23891641

[B3] Barghorn S. Biernat J. Mandelkow E. (2005). Purification of Recombinant Tau Protein and Preparation of Alzheimer-Paired Helical Filaments *In Vitro* . Methods Mol. Biol. 299, 035–052. 10.1385/1-59259-874-9:035 15980594

[B4] Barthelmé S. Tschumperlé D. (2019). Imager: an R Package for Image Processing Based on CImg. JOSS 4, 1012. 10.21105/joss.01012

[B5] Biernat J. Gustke N. Drewes G. Mandelkow E. Mandelkow E. (1993). Phosphorylation of Ser262 Strongly Reduces Binding of Tau to Microtubules: Distinction between PHF-like Immunoreactivity and Microtubule Binding. Neuron 11, 153–163. 10.1016/0896-6273(93)90279-z 8393323

[B6] Binder L. I. Frankfurter A. Rebhun L. I. (1985). The Distribution of Tau in the Mammalian Central Nervous System. J. Cell Biol. 101, 1371–1378. 10.1083/jcb.101.4.1371 3930508 PMC2113928

[B7] Bischof J. Randoll S.-J. Süßner N. Henne-Bruns D. Pinna L. A. Knippschild U. (2013). CK1δ Kinase Activity Is Modulated by Chk1-Mediated Phosphorylation. PLoS One 8, e68803. 10.1371/journal.pone.0068803 23861943 PMC3701638

[B8] Borchert N. Dieterich C. Krug K. Schütz W. Jung S. Nordheim A. (2010). Proteogenomics of Pristionchus Pacificus Reveals Distinct Proteome Structure of Nematode Models. Genome Res. 20, 837–846. 10.1101/gr.103119.109 20237107 PMC2877580

[B9] Briner A. Götz J. Polanco J. C. (2020). Fyn Kinase Controls Tau Aggregation *In Vivo* . Cell Rep. 32, 108045. 10.1016/j.celrep.2020.108045 32814048

[B10] Brion J. P. Hanger D. P. Couck A. M. Anderton B. H. (1991). A68 Proteins in Alzheimer's Disease Are Composed of Several Tau Isoforms in a Phosphorylated State Which Affects Their Electrophoretic Mobilities. Biochem. J. 279 (Pt 3), 831–836. 10.1042/bj2790831 1953678 PMC1151521

[B11] Buée L. Bussière T. Buée-Scherrer V. Delacourte A. Hof P. R. (2000). Tau Protein Isoforms, Phosphorylation and Role in Neurodegenerative disorders11These Authors Contributed Equally to This Work. Brain Res. Rev. 33, 95–130. 10.1016/s0165-0173(00)00019-9 10967355

[B12] Cheong J. K. Virshup D. M. (2011). Casein Kinase 1: Complexity in the Family. Int. J. Biochem. Cell Biol. 43, 465–469. 10.1016/j.biocel.2010.12.004 21145983

[B13] Chirita C. N. Congdon E. E. Yin H. Kuret J. (2005). Triggers of Full-Length Tau Aggregation: a Role for Partially Folded Intermediates. Biochemistry 44, 5862–5872. 10.1021/bi0500123 15823045

[B14] Choi S. H. Kim Y. H. Hebisch M. Sliwinski C. Lee S. D’Avanzo C. (2014). A Three-Dimensional Human Neural Cell Culture Model of Alzheimer's Disease. Nature 515, 274–278. 10.1038/nature13800 25307057 PMC4366007

[B15] Cox J. Mann M. (2008). MaxQuant Enables High Peptide Identification Rates, Individualized p.p.b.-range Mass Accuracies and Proteome-wide Protein Quantification. Nat. Biotechnol. 26, 1367–1372. 10.1038/nbt.1511 19029910

[B16] Cox J. Neuhauser N. Michalski A. Scheltema R. A. Olsen J. V. Mann M. (2011). Andromeda: a Peptide Search Engine Integrated into the MaxQuant Environment. J. Proteome Res. 10, 1794–1805. 10.1021/pr101065j 21254760

[B17] Dephoure N. Gould K. L. Gygi S. P. Kellogg D. R. (2013). Mapping and Analysis of Phosphorylation Sites: a Quick Guide for Cell Biologists. MBoC 24, 535–542. 10.1091/mbc.e12-09-0677 23447708 PMC3583658

[B18] Derkinderen P. Scales T. M. E. Hanger D. P. Leung K.-Y. Byers H. L. Ward M. A. (2005). Tyrosine 394 Is Phosphorylated in Alzheimer's Paired Helical Filament Tau and in Fetal Tau with C-Abl as the Candidate Tyrosine Kinase. J. Neurosci. 25, 6584–6593. 10.1523/JNEUROSCI.1487-05.2005 16014719 PMC6725430

[B19] Despres C. Byrne C. Qi H. Cantrelle F.-X. Huvent I. Chambraud B. (2017). Identification of the Tau Phosphorylation Pattern that Drives its Aggregation. Proc. Natl. Acad. Sci. U.S.A. 114, 9080–9085. 10.1073/pnas.1708448114 28784767 PMC5576827

[B20] Ghoshal N. Smiley J. F. DeMaggio A. J. Hoekstra M. F. Cochran E. J. Binder L. I. (1999). A New Molecular Link between the Fibrillar and Granulovacuolar Lesions of Alzheimer's Disease. Am. J. Pathology 155, 1163–1172. 10.1016/S0002-9440(10)65219-4 PMC1867028 10514399

[B21] Goedert M. Spillantini M. G. Jakes R. Rutherford D. Crowther R. A. (1989). Multiple Isoforms of Human Microtubule-Associated Protein Tau: Sequences and Localization in Neurofibrillary Tangles of Alzheimer's Disease. Neuron 3, 519–526. 10.1016/0896-6273(89)90210-9 2484340

[B22] Grundke-Iqbal I. Iqbal K. Tung Y. C. Quinlan M. Wisniewski H. M. Binder L. I. (1986). Abnormal Phosphorylation of the Microtubule-Associated Protein Tau (Tau) in Alzheimer Cytoskeletal Pathology. Proc. Natl. Acad. Sci. U.S.A. 83, 4913–4917. 10.1073/pnas.83.13.4913 3088567 PMC323854

[B23] Hanger D. P. Anderton B. H. Noble W. (2009). Tau Phosphorylation: the Therapeutic Challenge for Neurodegenerative Disease. Trends Mol. Med. 15, 112–119. 10.1016/j.molmed.2009.01.003 19246243

[B24] Hanger D. P. Betts J. C. Loviny T. L. F. Blackstock W. P. Anderton B. H. (1998). New Phosphorylation Sites Identified in Hyperphosphorylated Tau (Paired Helical Filament-Tau) from Alzheimer's Disease Brain Using Nanoelectrospray Mass Spectrometry. J. Neurochem. 71, 2465–2476. 10.1046/j.1471-4159.1998.71062465.x 9832145

[B25] Hanger D. P. Byers H. L. Wray S. Leung K.-Y. Saxton M. J. Seereeram A. (2007). Novel Phosphorylation Sites in Tau from Alzheimer Brain Support a Role for Casein Kinase 1 in Disease Pathogenesis. J. Biol. Chem. 282, 23645–23654. 10.1074/jbc.M703269200 17562708

[B26] Ianes C. Xu P. Werz N. Meng Z. Henne-Bruns D. Bischof J. (2016). CK1δ Activity Is Modulated by CDK2/E- and CDK5/p35-Mediated Phosphorylation. Amino Acids 48, 579–592. 10.1007/s00726-015-2114-y 26464264

[B27] Illenberger S. Zheng-Fischhöfer Q. Preuss U. Stamer K. Baumann K. Trinczek B. (1998). The Endogenous and Cell Cycle-dependent Phosphorylation of Tau Protein in Living Cells: Implications for Alzheimer's Disease. MBoC 9, 1495–1512. 10.1091/mbc.9.6.1495 9614189 PMC25374

[B28] Kim Y. H. Choi S. H. D'Avanzo C. Hebisch M. Sliwinski C. Bylykbashi E. (2015). A 3D Human Neural Cell Culture System for Modeling Alzheimer's Disease. Nat. Protoc. 10, 985–1006. 10.1038/nprot.2015.065 26068894 PMC4499058

[B29] Kimura T. Ishiguro K. Hisanaga S.-I. (2014). Physiological and Pathological Phosphorylation of Tau by Cdk5. Front. Mol. Neurosci. 7, 65. 10.3389/fnmol.2014.00065 25076872 PMC4097945

[B30] Kinoshita E. Kinoshita-Kikuta E. Takiyama K. Koike T. (2006). Phosphate-binding Tag, a New Tool to Visualize Phosphorylated Proteins. Mol. Cell. Proteomics 5, 749–757. 10.1074/mcp.T500024-MCP200 16340016

[B31] Knippschild U. KrÃ¼ger M. Richter J. Xu P. GarcÃ-a-Reyes B. Peifer C. (2014). The CK1 Family: Contribution to Cellular Stress Response and its Role in Carcinogenesis. Front. Oncol. 4, 96. 10.3389/fonc.2014.00096 24904820 PMC4032983

[B32] Knippschild U. Milne D. M. Campbell L. E. DeMaggio A. J. Christenson E. Hoekstra M. F. (1997). p53 Is Phosphorylated *In Vitro* and *In Vivo* by the Delta and Epsilon Isoforms of Casein Kinase 1 and Enhances the Level of Casein Kinase 1 Delta in Response to Topoisomerase-Directed Drugs. Oncogene 15, 1727–1736. 10.1038/sj.onc.1201541 9349507

[B33] Kosik K. S. Joachim C. L. Selkoe D. J. (1986). Microtubule-associated Protein Tau (Tau) Is a Major Antigenic Component of Paired Helical Filaments in Alzheimer Disease. Proc. Natl. Acad. Sci. U.S.A. 83, 4044–4048. 10.1073/pnas.83.11.4044 2424016 PMC323662

[B34] Krismer K. Bernwinkler T. Ehrenberger T. (2021). Scan Protein for Motifs - Scansite 4 . https://scansite4.mit.edu/#scanProtein (Accessed March 15, 2022).

[B35] Ksiezak-Reding H. Pyo H. K. Feinstein B. Pasinetti G. M. (2003). Akt/PKB Kinase Phosphorylates Separately Thr212 and Ser214 of Tau Protein *In Vitro* . Biochimica Biophysica Acta (BBA) - Mol. Basis Dis. 1639, 159–168. 10.1016/j.bbadis.2003.09.001 14636947

[B36] Kumar A. Dogra S. (2008). Neuropathology and Therapeutic Management of Alzheimer's Disease - an Update. Drugs Fut. 33, 433. 10.1358/dof.2008.033.05.1192677

[B37] Kuret J. Johnson G. S. Cha D. Christenson E. R. DeMaggio A. J. Hoekstra M. F. (1997). Casein Kinase 1 Is Tightly Associated with Paired-Helical Filaments Isolated from Alzheimer's Disease Brain. J. Neurochem. 69, 2506–2515. 10.1046/j.1471-4159.1997.69062506.x 9375684

[B38] Lee G. Neve R. L. Kosik K. S. (1989). The Microtubule Binding Domain of Tau Protein. Neuron 2, 1615–1624. 10.1016/0896-6273(89)90050-0 2516729

[B39] Lee G. Thangavel R. Sharma V. M. Litersky J. M. Bhaskar K. Fang S. M. (2004). Phosphorylation of Tau by Fyn: Implications for Alzheimer's Disease. J. Neurosci. 24, 2304–2312. 10.1523/JNEUROSCI.4162-03.2004 14999081 PMC6730442

[B40] Li G. Yin H. Kuret J. (2004). Casein Kinase 1 Delta Phosphorylates Tau and Disrupts its Binding to Microtubules. J. Biol. Chem. 279, 15938–15945. 10.1074/jbc.M314116200 14761950

[B41] Liachko N. F. McMillan P. J. Strovas T. J. Loomis E. Greenup L. Murrell J. R. (2014). The Tau Tubulin Kinases TTBK1/2 Promote Accumulation of Pathological TDP-43. PLoS Genet. 10, e1004803. 10.1371/journal.pgen.1004803 25473830 PMC4256087

[B42] Liu F. Li B. Tung E.-J. Grundke-Iqbal I. Iqbal K. Gong C.-X. (2007). Site-specific Effects of Tau Phosphorylation on its Microtubule Assembly Activity and Self-Aggregation. Eur. J. Neurosci. 26, 3429–3436. 10.1111/j.1460-9568.2007.05955.x 18052981 PMC2262108

[B43] Liu S. Zhang C. Campbell J. L. Zhang H. Yeung K. K.-C. Han V. K. M. (2005). Formation of Phosphopeptide-Metal Ion Complexes in Liquid Chromatography/electrospray Mass Spectrometry and Their Influence on Phosphopeptide Detection. Rapid Commun. Mass Spectrom. 19, 2747–2756. 10.1002/rcm.2105 16136520

[B44] Llorens-MarÃ- tin M. a. Jurado J. HernÃ¡ndez F. l. Ãvila J. (2014). GSK-3Î², a Pivotal Kinase in Alzheimer Disease. Front. Mol. Neurosci. 7, 46. 10.3389/fnmol.2014.00046 24904272 PMC4033045

[B45] Luque-Garcia J. L. Neubert T. A. (2009). On-membrane Tryptic Digestion of Proteins for Mass Spectrometry Analysis. Methods Mol. Biol. 536, 331–341. 10.1007/978-1-59745-542-8_35 19378072 PMC3757930

[B46] Matenia D. Mandelkow E.-M. (2009). The Tau of MARK: a Polarized View of the Cytoskeleton. Trends Biochem. Sci. 34, 332–342. 10.1016/j.tibs.2009.03.008 19559622

[B47] Matsuo E. S. Shin R.-W. Billingsley M. L. van deVoorde A. O'Connor M. Trojanowski J. Q. (1994). Biopsy-derived Adult Human Brain Tau Is Phosphorylated at Many of the Same Sites as Alzheimer's Disease Paired Helical Filament Tau. Neuron 13, 989–1002. 10.1016/0896-6273(94)90264-X 7946342

[B48] Mendoza J. Sekiya M. Taniguchi T. Iijima K. M. Wang R. Ando K. (2013). Global Analysis of Phosphorylation of Tau by the Checkpoint Kinases Chk1 and Chk2*In Vitro* . J. Proteome Res. 12, 2654–2665. 10.1021/pr400008f 23550703 PMC3757556

[B49] Meng Z. Bischof J. Ianes C. Henne-Bruns D. Xu P. Knippschild U. (2016). CK1δ Kinase Activity Is Modulated by Protein Kinase C α (PKCα)-Mediated Site-specific Phosphorylation. Amino Acids 48, 1185–1197. 10.1007/s00726-015-2154-3 26803658

[B50] Meng Z. Böhm T. Xu P. Henne-Bruns D. Peifer C. Witt L. (2019). Kinase Activity of Casein Kinase 1 Delta (CK1δ) Is Modulated by Protein Kinase C α (PKCα) by Site-specific Phosphorylation within the Kinase Domain of CK1δ. Biochimica Biophysica Acta (BBA) - Proteins Proteomics 1867, 710–721. 10.1016/j.bbapap.2019.05.004 31096047

[B51] Morishima-Kawashima M. Hasegawa M. Takio K. Suzuki M. Yoshida H. Titani K. (1995a). Proline-directed and Non-proline-directed Phosphorylation of PHF-Tau. J. Biol. Chem. 270, 823–829. 10.1074/jbc.270.2.823 7822317

[B52] Morishima-Kawashima M. Hasegawa M. Takio K. Suzuki M. Yoshida H. Watanabe A. (1995b). Hyperphosphorylation of Tau in PHF. Neurobiol. Aging 16, 365–371. 10.1016/0197-4580(95)00027-C 7566346

[B53] Noble W. Hanger D. P. Miller C. C. J. Lovestone S. (2013). The Importance of Tau Phosphorylation for Neurodegenerative Diseases. Front. Neurol. 4, 83. 10.3389/fneur.2013.00083 23847585 PMC3696910

[B54] Pickhardt M. Gazova Z. von Bergen M. Khlistunova I. Wang Y. Hascher A. (2005). Anthraquinones Inhibit Tau Aggregation and Dissolve Alzheimer's Paired Helical Filaments *In Vitro* and in Cells. J. Biol. Chem. 280, 3628–3635. 10.1074/jbc.M410984200 15525637

[B55] Rappsilber J. Mann M. Ishihama Y. (2007). Protocol for Micro-purification, Enrichment, Pre-fractionation and Storage of Peptides for Proteomics Using StageTips. Nat. Protoc. 2, 1896–1906. 10.1038/nprot.2007.261 17703201

[B56] Roth A. Gärtner F. Mayer K. Beyrle J. König I. Knippschild U. (2021a). CK1δ-Derived Peptides as Novel Tools Inhibiting the Interactions between CK1δ and APP695 to Modulate the Pathogenic Metabolism of APP. Ijms 22, 6423. 10.3390/ijms22126423 34203978 PMC8232658

[B57] Roth A. Gihring A. Göser F. Peifer C. Knippschild U. Bischof J. (2021b). Assessing the Inhibitory Potential of Kinase Inhibitors *In Vitro*: Major Pitfalls and Suggestions for Improving Comparability of Data Using CK1 Inhibitors as an Example. Molecules 26, 4898. 10.3390/molecules26164898 34443486 PMC8401859

[B58] Rudrabhatla P. Jaffe H. Pant H. C. (2011). Direct Evidence of Phosphorylated Neuronal Intermediate Filament Proteins in Neurofibrillary Tangles (NFTs): Phosphoproteomics of Alzheimer's NFTs. FASEB J. 25, 3896–3905. 10.1096/fj.11-181297 21828286 PMC3205835

[B59] Schmitt M. Sinnberg T. Nalpas N. C. Maass A. Schittek B. Macek B. (2019). Quantitative Proteomics Links the Intermediate Filament Nestin to Resistance to Targeted BRAF Inhibition in Melanoma Cells. Mol. Cell. Proteomics 18, 1096–1109. 10.1074/mcp.RA119.001302 30890564 PMC6553926

[B60] Schwab C. DeMaggio A. J. Ghoshal N. Binder L. I. Kuret J. McGeer P. L. (2000). Casein Kinase 1 Delta Is Associated with Pathological Accumulation of Tau in Several Neurodegenerative Diseases. Neurobiol. Aging 21, 503–510. 10.1016/s0197-4580(00)00110-x 10924763

[B61] Solari F. A. Dell'Aica M. Sickmann A. Zahedi R. P. (2015). Why Phosphoproteomics Is Still a Challenge. Mol. Biosyst. 11, 1487–1493. 10.1039/C5MB00024F 25800119

[B62] Stöter M. Krüger M. Banting G. Henne-Bruns D. Knippschild U. (2014). Microtubules Depolymerization Caused by the CK1 Inhibitor IC261 May Be Not Mediated by CK1 Blockage. PLoS One 9, e100090. 10.1371/journal.pone.0100090 24937750 PMC4061085

[B63] Thornton C. Bright N. J. Sastre M. Muckett P. J. Carling D. (2011). AMP-activated Protein Kinase (AMPK) Is a Tau Kinase, Activated in Response to Amyloid β-peptide Exposure. Biochem. J. 434, 503–512. 10.1042/BJ20101485 21204788

[B64] Tomizawa K. Omori A. Ohtake A. Sato K. Takahashi M. (2001). Tau-tubulin Kinase Phosphorylates Tau at Ser-208 and Ser-210, Sites Found in Paired Helical Filament-Tau. FEBS Lett. 492, 221–227. 10.1016/S0014-5793(01)02256-6 11257498

[B65] van der Geer P. Hunter T. (1994). Phosphopeptide Mapping and Phosphoamino Acid Analysis by Electrophoresis and Chromatography on Thin-Layer Cellulose Plates. Electrophoresis 15, 544–554. 10.1002/elps.1150150173 8055882

[B66] Vandermarliere E. Mueller M. Martens L. (2013). Getting Intimate with Trypsin, the Leading Protease in Proteomics. Mass Spec. Rev. 32, 453–465. 10.1002/mas.21376 23775586

[B67] Weingarten M. D. Lockwood A. H. Hwo S. Y. Kirschner M. W. (1975). A Protein Factor Essential for Microtubule Assembly. Proc. Natl. Acad. Sci. U.S.A. 72, 1858–1862. 10.1073/pnas.72.5.1858 1057175 PMC432646

[B68] Wolozin B. L. Pruchnicki A. Dickson D. W. Davies P. (1986). A Neuronal Antigen in the Brains of Alzheimer Patients. Science 232, 648–650. 10.1126/science.3083509 3083509

[B69] Xian J. Bu F. Wang Y. Long F. Zhang Z. Wu C. (2021). A Rationale for Drug Design provided by Co-Crystal Structure of IC261 in Complex with Tubulin. Molecules 26, 946. 10.3390/molecules26040946 33579052 PMC7916759

[B70] Xu J. Sato S. Okuyama S. Swan R. J. Jacobsen M. T. Strunk E. (2010). Tau‐tubulin Kinase 1 Enhances Prefibrillar Tau Aggregation and Motor Neuron Degeneration in P301L FTDP‐17 Tau‐mutant Mice. FASEB J. 24, 2904–2915. 10.1096/fj.09-150144 20354135

[B71] Yasojima K. Kuret J. DeMaggio A. J. McGeer E. McGeer P. L. (2000). Casein Kinase 1 Delta mRNA Is Upregulated in Alzheimer Disease Brain. Brain Res. 865, 116–120. 10.1016/s0006-8993(00)02200-9 10814741

[B72] Zheng-Fischhöfer Q. Biernat J. Mandelkow E.-M. Illenberger S. Godemann R. Mandelkow E. (1998). Sequential Phosphorylation of Tau by Glycogen Synthase Kinase-3beta and Protein Kinase A at Thr212 and Ser214 Generates the Alzheimer-specific Epitope of Antibody AT100 and Requires a Paired-helical-filament-like Conformation. Eur. J. Biochem. 252, 542–552. 10.1046/j.1432-1327.1998.2520542.x 9546672

