# Supplementary material for: Comprehensive Characterization of CK1δ-Mediated Tau Phosphorylation in Alzheimer’s Disease
Source: Front Mol Biosci. 2022 Jun 27;9:872171. doi: 10.3389/fmolb.2022.872171 (PMC9531328; doi:10.3389/fmolb.2022.872171)
Supplement: Supplementary file 2 [file DataSheet1.docx]

Supplementary Material

# Supplementary Data

## Supplementary Tables

Supplementary Table 1: Sequences of tau441-specific primers used for cloning and site-directed mutagenesis.

| **Construct** | **Primer** | **Sequence (5’ 🡪 3’)** |
| --- | --- | --- |
| pET28a(+)tau441^1-155^ | F1.1 | AACCCCGCGCTAAGCAGCCCCGC |
|  | F1.2 | GCAATTTTGGTTTTGCCATCGGC |
| pET28a(+)tau441^243-441^ | F2.1 | CTGCAGACCGCCCCGGTT |
|  | F2.2 | GGATCCGCGACCCATTTGC |
| pET28a(+)tau441^156-242^ | GA1 | AGCAAATGGGTCGCGGATCCGGTGCAGCCCCGCCTGGT |
|  | GA2 | TGGTGGTGGTGGTGCTCGAGTTAGCGGCTTTTGGCACTACTCGGAC |
| pET28a(+)tau441^S68A^ | P1.1 | TGATGCAAAAGCCACCCCGACCG |
|  | P1.2 | CTGGTTTCACTACCCGGT |
| pET28a(+)tau441^S68A+T69A+T71A^ | P1.3 | GGCAGCAGAAGATGTGACCGCC |
|  | P1.4 | GGTGCGGCTTTTGCATCACTGGTTTC |
| pET28a(+)tau441^S202A+T205A^ | P2.1 | CAGCCCGGGTGCACCGGGCGCAC |
|  | P2.2 | CCCGGGCTGCTATAACCACTGCG |
| pET28a(+)tau441^S198A+S199A+S202A+T205A^ | P2.3 | CAGTGGTTATGCAGCACCGGGTGCACCG |
|  | P2.4 | CACTGCGGTCACCGCTTTTC |
| pET28a(+)tau441^T212A+S214A^ | P3.1 | TCGTGCACCGGCACTGCCGACCCCG |
|  | P3.2 | CTACGGCTACCCGGGGTG |
| pET28a(+)tau441^T212A+S214A+T217A+T220A^ | P3.3 | GGCACCGCCTGCACGTGAACCGAAAAAAG |
|  | P3.4 | GGCAGGGCCGGTGCACGA |
| pET28a(+)tau441^S289A^ | P4.1 | TAACGTTCAGGCGAAATGTGGTAGCAAAG |
|  | P4.2 | CTCAGGTCCAGTTTCTTATTAATAATC |
| pET28a(+)tau441^S412A+S413A^ | P5.1 | GAGTAATGTTGCGGCAACCGGCAGCATTGATATGGTTGATAGCCC |
|  | P5.2 | AGATGACGCGGGCTGGTA |
| pET28a(+)tau441^S412A+S413A+T414A+S416A^ | P5.3 | CGCAATTGATATGGTTGATAGCCC |
|  | P5.4 | CCTGCTGCCGCAACATTACTCAG |
| pET28a(+)tau441^S409A+S412A+S413A +T414A+S416A^ | P5.5 | GCGTCATCTGGCCAATGTTGCGGCAGCAG |
|  | P5.6 | GGGCTGGTATCGCCGCTC |
| pET28a(+)tau441^S422A+S427A^ | P6.1 | CTGGCAGCACTGGCAGATGAAGTGAGC |
|  | P6.2 | CTGCGGTGCATCAACCATATCAATGCTGC |

## Supplementary Figures


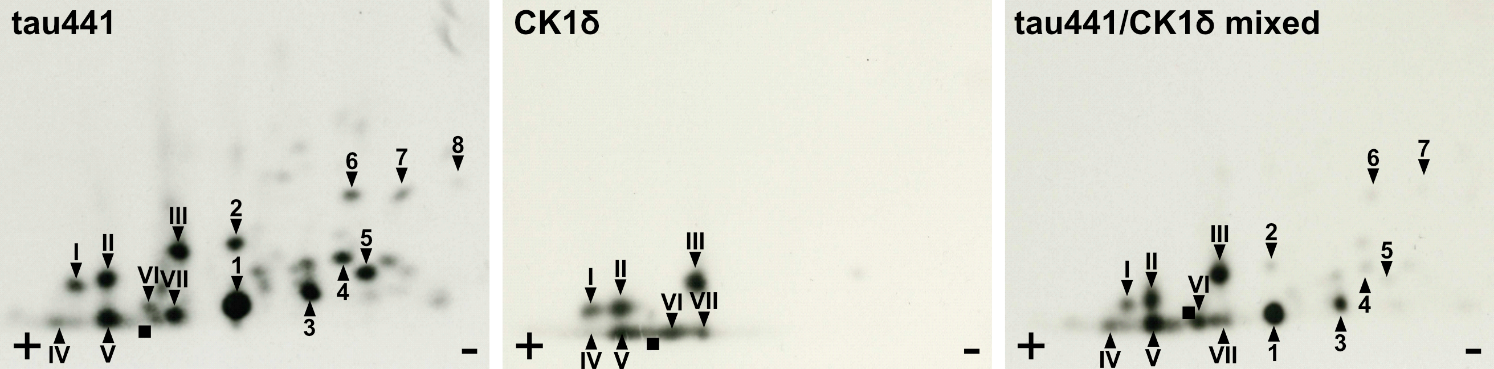


Supplementary Figure 1: Phosphopeptide analysis of CK1δ-phosphorylated tau441 and autophosphorylated CK1δ. Phosphorylated tau441 and autophosphorylated CK1δ were analyzed by two-dimensional phosphopeptide analysis. Numbers indicate major phosphorylated peptides, which can be assigned to wild type tau441. Phosphopeptides, that are exclusively phosphorylated in the fragment are indicated with letters. Roman numbers indicated phosphopeptides that correspond to autophosphorylated CK1δ. ■: loading point of samples, +: anode, ‑: cathode.


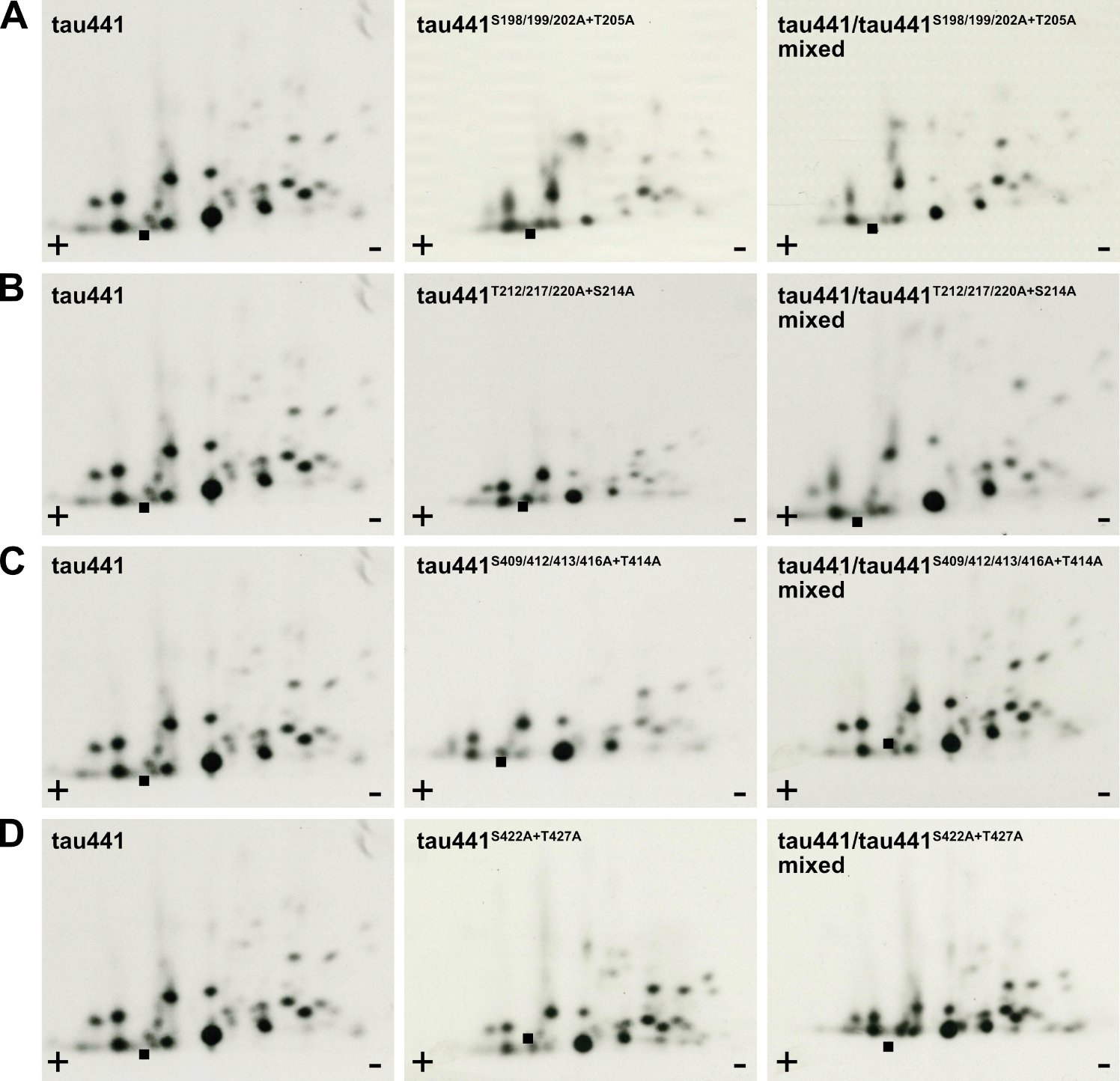


Supplementary Figure 2: Phosphopeptide analysis of CK1δ-phosphorylated tau441 phosphomutants. Tau441 phosphomutants (A) tau441^S198A+S199A+S202A+T205A^, (B) tau441^T212A+S214A+T217A+T220A^, (C) tau441^S409A+S412A+S413A+T414A+S416A^ and (D) tau441^S422A+T427A^ were phosphorylated by CK1δ *in vitro* and analyzed by two-dimensional phosphopeptide analysis. ■: loading point of samples, +: anode, ‑: cathode.


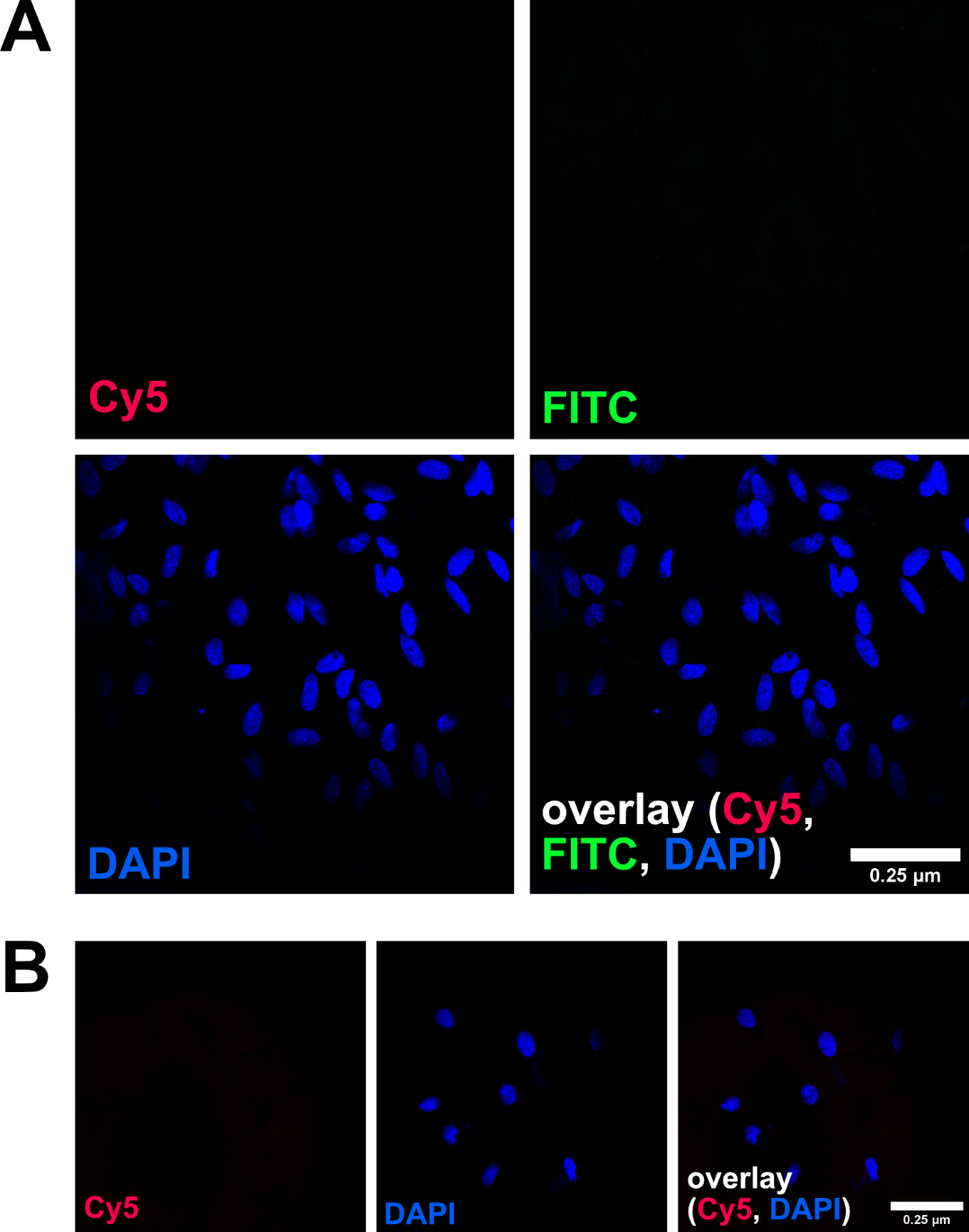


Supplementary Figure 3: Co-immunofluorescence of tau and CK1δ in differentiated hNPCs. Representative image of negative controls in which primary antibodies (anti-tau antibody and anti-CK1δ antibody) were omitted. Cells were stained with DAPI to show to cell nucleus (blue). Scale bar: 0.25 µm.
